# Novel Cyclopentaquinoline and Acridine Analogs as Multifunctional, Potent Drug Candidates in Alzheimer’s Disease

**DOI:** 10.3390/ijms23115876

**Published:** 2022-05-24

**Authors:** Karolina Maciejewska, Kamila Czarnecka, Paweł Kręcisz, Dorota Niedziałek, Grzegorz Wieczorek, Robert Skibiński, Paweł Szymański

**Affiliations:** 1 Department of Pharmaceutical Chemistry, Drug Analyses and Radiopharmacy, Faculty of Pharmacy, Medical University of Lodz, 90-151 Lodz, Poland; karolina.maciejewska@stud.umed.lodz.pl (K.M.); kamila.czarnecka@umed.lodz.pl (K.C.); pawel.krecisz@stud.umed.lodz.pl (P.K.); 2 Institute of Biochemistry and Biophysics, Polish Academy of Sciences, 02-106 Warsaw, Poland; dorotanka@gmail.com (D.N.); wieczorek.grzegorz@gmail.com (G.W.); 3 Department of Medicinal Chemistry, Faculty of Pharmacy, Medical University of Lublin, 20-090 Lublin, Poland; robertskibinski@umlub.pl; 4 Department of Radiobiology and Radiation Protection, Military Institute of Hygiene and Epidemiology, 01-163 Warsaw, Poland

**Keywords:** Alzheimer’s disease, multitarget small molecules, biological activity

## Abstract

A series of new cyclopentaquinoline derivatives with 9-acridinecarboxylic acid and a different alkyl chain length were synthesized, and their ability to inhibit cholinesterases was evaluated. All designed compounds, except derivative **3f**, exhibited a selectivity for butyrylcholinesterase (BuChE) with IC_50_ values ranging from 103 to 539 nM. The **3b** derivative revealed the highest inhibitory activity towards BuChE (IC_50_ = 103.73 nM) and a suitable activity against AChE (IC_50_ = 272.33 nM). The **3f** derivative was the most active compound to AChE (IC_50_ = 113.34 nM) with satisfactory activity towards BuChE (IC_50_ = 203.52 nM). The potential hepatotoxic effect was evaluated for both **3b** and **3f** compounds. The **3b** and **3f** potential antioxidant activity was measured using the ORAC-FL method. The **3b** and **3f** derivatives revealed a significantly higher antioxidant potency, respectively 35 and 25 higher than tacrine. Theoretical, physicochemical, and pharmacokinetic properties were calculated using ACD Labs Percepta software. Molecular modeling and kinetic study were used to reveal the mechanism of cholinesterase inhibition in the most potent compounds: **3b** and **3f**.

## 1. Introduction

Dementia has become a relevant health problem with a significant socio-economic impact worldwide. Nowadays, 50 million people are suffering from dementia, and by 2050 this number will have doubled [1]. Alzheimer’s disease (AD) is regarded as the prevailing cause of dementia, consisting of 60–70% of all cases [2].

There are two known forms of Alzheimer’s diseases: sporadic (SAD), or late-onset form of AD (LOAD), which reveals itself after 65 years of age and dominates in statistics of AD [3], and the hereditary ‘familial’ form (FAD). FAD is characterized by an early onset of the disease, correlated with a mutation of the genes encoding amyloid precursor protein (APP) and presenilin (PSEN1 and PSEN2) [4,5]. However, both forms of Alzheimer’s disease share similar pathological processes leading to specific molecular manifestations, such as extracellular amyloid β (Aβ) deposition, hyperphosphorylation tau protein—and its intracellular accumulation—oxidative stress, neuro-inflammation, mitochondrial impairment, and ion metabolism disorders [6].

The development of medicine, particularly neuroimaging techniques, has enabled a faster and more accurate diagnostic for Alzheimer’s disease and other forms of dementia. Alzheimer’s disease neuroimaging includes structural and functional magnetic resonance imaging (MRI) [7], fluorodeoxyglucose PET, and Aβ and tau PET uptake [7,8].

Over the years, scientists have proposed several hypotheses for explaining the development of Alzheimer’s disease. Acetylcholine (ACh) is an essential neurotransmitter involved in cognitive processes. The cholinergic hypothesis, including the cholinergic neurons impairment and synaptic acetylcholine deficit, represents the indisputable cause of AD [9]. The disruption of cholinergic neurotransmission in AD is manifested by the deterioration of memory, learning processes, and the presence of neurobehavioral symptoms, such as aggression and agitation [9,10]. Cholinesterases play a crucial role in the cholinergic hypothesis. AChE and BuChE hydrolyze ACh in the synaptic cleft regulating the level of ACh in neurons [9]. In Alzheimer’s disease, the levels of both cholinesterases are increased, which is responsible for the ACh level decrease. A decreased level of ACh directly contributes to the impairment of cognitive function and neuroinflammation [11]. Moreover, studies have shown that both cholinesterases bind to Aβ to form complexes that exhibit a greater neurotoxicity and stability than unbound Aβ [12,13]. This is a result of the homology between the C-terminal fragment of AChE and the N-terminal fragment of Aβ [12,14]. AChE-Aβ complexes cause an uncontrolled Ca^2+^ influx into the neuron’s interior resulting in mitochondrial dysfunction and the loss of membrane potential [13]. Although the cholinergic hypothesis loses importance in the pathogenesis of Alzheimer’s disease in favor of β-amyloid, cholinergic-based strategies remain the most common approach in the treatment of AD and other forms of dementia [15].

The amyloid hypothesis is now regarded as the main causal factor in the progression of AD [16]. Studies have revealed that Aβ significantly disrupts synaptic plasticity and induces the production of reactive oxygen species (ROS), which contribute to the impairment of mitochondrial function and neuro-inflammation. Naturally, the neurotoxicity of Aβ is multifactorial and affects many processes, including the neuronal cell structure, leading to the aggregation and accumulation of Aβ fibrils as extracellular plaques [4,17].

Another pivotal protein in the development of AD neurodegeneration is the tau protein [4]. Novel studies suggest that tau pathology may be an initiating factor in sporadic AD. Similarly to Aβ, after excessive post-translational hyperphosphorylation, the tau protein aggregates forming intracellular deposits, or ‘fibrils’ (neurofibrillary tangles—NFTs) [4]. However, the presence of Aβ and NFTs is not exclusively synonymous with Alzheimer’s disease. Especially, abnormalities in tau production and the metabolism have been identified in different neurodegenerative disorders [18].

Nowadays, only four drugs are approved for the treatment of Alzheimer’s disease: three acetylcholinesterase inhibitors (donepezil, galantamine, and rivastigmine) and memantine, an antagonist of N-methyl-D-aspartate (NMDA) receptors [10]. Acetylcholinesterase inhibitors bring symptomatic relief in mild to moderate AD, while memantine is mostly used in moderate and severe dementia. Combination therapy using acetylcholinesterase inhibitors and memantine demonstrated higher clinical efficacy compared to monotherapy [19]. Recently developed by Biogen Aducanumab, a human monoclonal antibody biding Aβ fibrils and soluble oligomers is the first approved drug directly targeting Aβ [20,21,22].

The priority of Alzheimer’s disease research is to develop a disease-modifying treatment that can be used in the early stage of AD, unlike currently used drugs that provide only symptomatic relief [19,23]. The pathogenesis of AD is considered multifactorial. Therefore, therapeutic strategies that will act on several biological targets simultaneously may be a promising approach to modify the course of AD. The multi-target-directed ligands (MTDLs) approach inspires researchers to develop hybrid molecules consisting of subunits with a proven biological activity for different molecular targets [15].

Following the MTDLs strategy, and the encouraged positive results of previous work Chufarova et al. [24], we synthesized a new series of cyclopentaquinoline derivatives with acridinecarboxylic acid. The compounds were evaluated as AChE and BuChE inhibitors using Ellman’s method. Moreover, antioxidant activity using the ORAC-FL method was investigated. Then, in vitro studies of hepatotoxicity were performed. Molecular modelling was used to evaluate the mechanism of cholinesterase inhibition for synthesized compounds, and an ADMET analysis was performed to predict the pharmacokinetic properties of the compounds.

## 2. Results

### 2.1. Chemistry

**Scheme 1:**
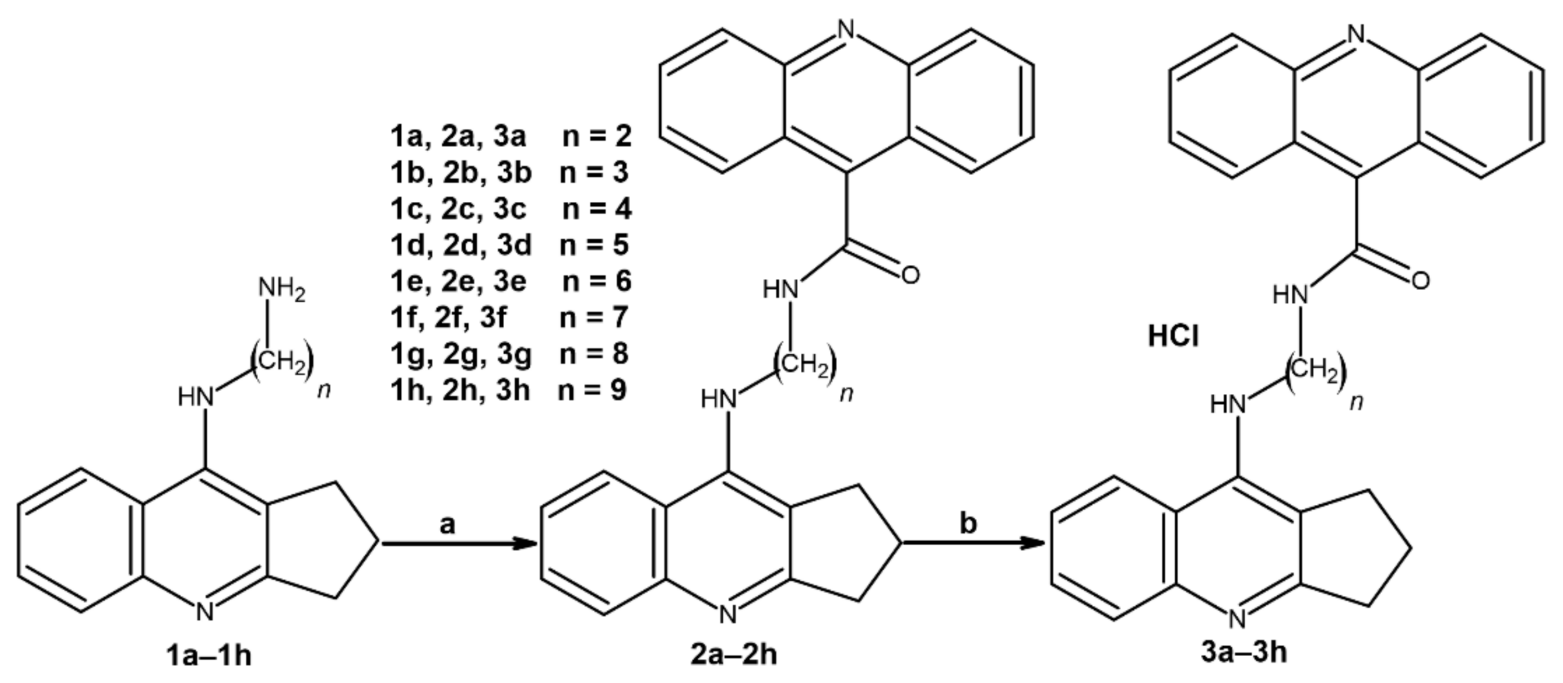
Synthetic pathway of compounds **2a**–**2h** and **3a**–**3h**. Chemical reagents: (**a**) 3,5-dichlorobenzoic acid, CDMT, *N*-methylmorpholine, THF; (**b**) HCl/ether.

The novel cyclopentaquinoline derivatives with acridine acid were synthesized according to Scheme 1. The substrates for the first step of synthesis, compounds **1a**–**1h**, were synthesized according to the previously described method [25,26]. The **1a**–**1h** compounds were combined with 9-acridinecarboxylic acid to form an amide bond in the presence of tetrahydrofuran (THF), N-methylmorpholine (NMM), and 2-chloro-4,6-dimethoxy-1,3,5-triazine (CDMT). The formation of the amide bond requires the activation of the carboxylic group by CDMT in the presence of the tertiary amine base, NMM. The reaction mechanism is based on two substitutions in the triazine ring [27]. Firstly, chlorine is substituted by NMM resulting in quaternary ammonium salt. Then, the carboxylate ion substitutes the quaternary ammonium salt with the generation of highly reactive triazine activated ester and releasing NMM, and thus promoting the amide coupling [27,28]. Finally, the activated ester undergoes nucleophilic substitution by amine resulting in **2a**–**2h** compounds and 1-hydroxy-3,5-dimetoxytriazine as a byproduct. In the second step of synthesis, **2a**–**2h** compounds were transformed into hydrochlorides using diethyl ether saturated with hydrogen chloride.

The chemical structures of final compounds were verified and proven by ^1^H and ^13^C NMR, ESI-MS, HRMS, and IR. In the experimental section we explain, in detail, the chemical shifts of the protons.

### 2.2. Biological Evaluation

#### 2.2.1. In Vitro Inhibition Studies AChE and BuChE

Ellman’s assay [29,30] was used to assess the inhibitory activity of the newly synthesized compounds towards AChE and BuChE. The obtained IC_50_ values—reflecting the compound’s concentration causing the 50% inhibition of the cholinesterases’ activity—for the synthesized compounds and references are summarized in Table 1. Most of the new derivatives exhibited a satisfactory inhibitory activity for both AChE and BuChE. Compounds **3f** with a longer alkyl chain revealed the highest inhibitory activity for AChE (IC_50_ = 113.34 nM). On the other hand, the most effective compound for BuChE was compound **3b** (IC_50_ = 103.73 nM) with a short alkyl chain. Despite derivative **3f**, all synthesized derivatives exhibited higher selectivity for BuChE rather than AChE. Compounds **3b** and **3f** were chosen for further experiments.

#### 2.2.2. Kinetic Characterization of AChE and BuChE Inhibition

**Figure 1:**
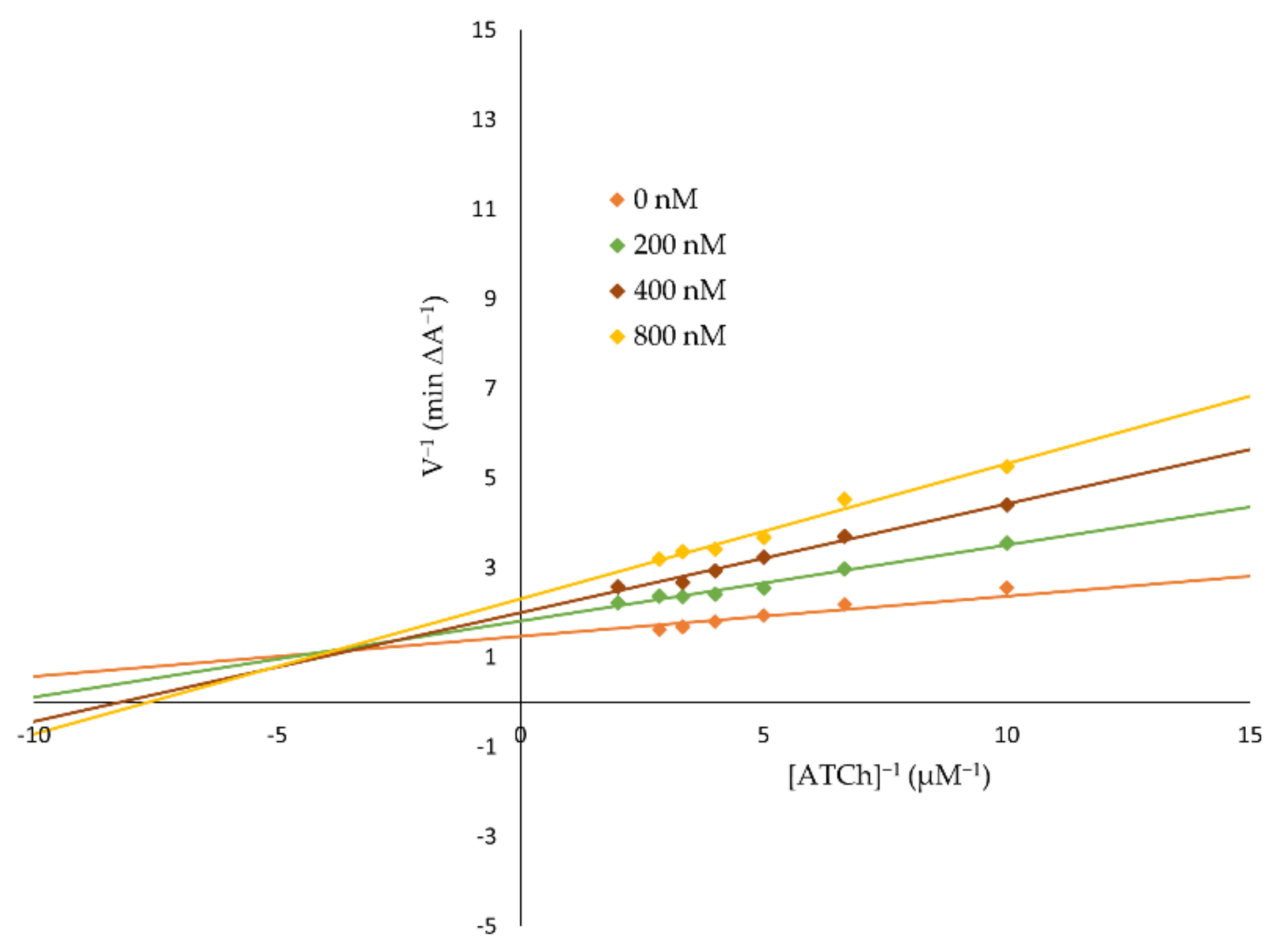
A Lineweaver-Burk plot presenting a mixed-type of AChE inhibition by **3f**.

**Figure 2:**
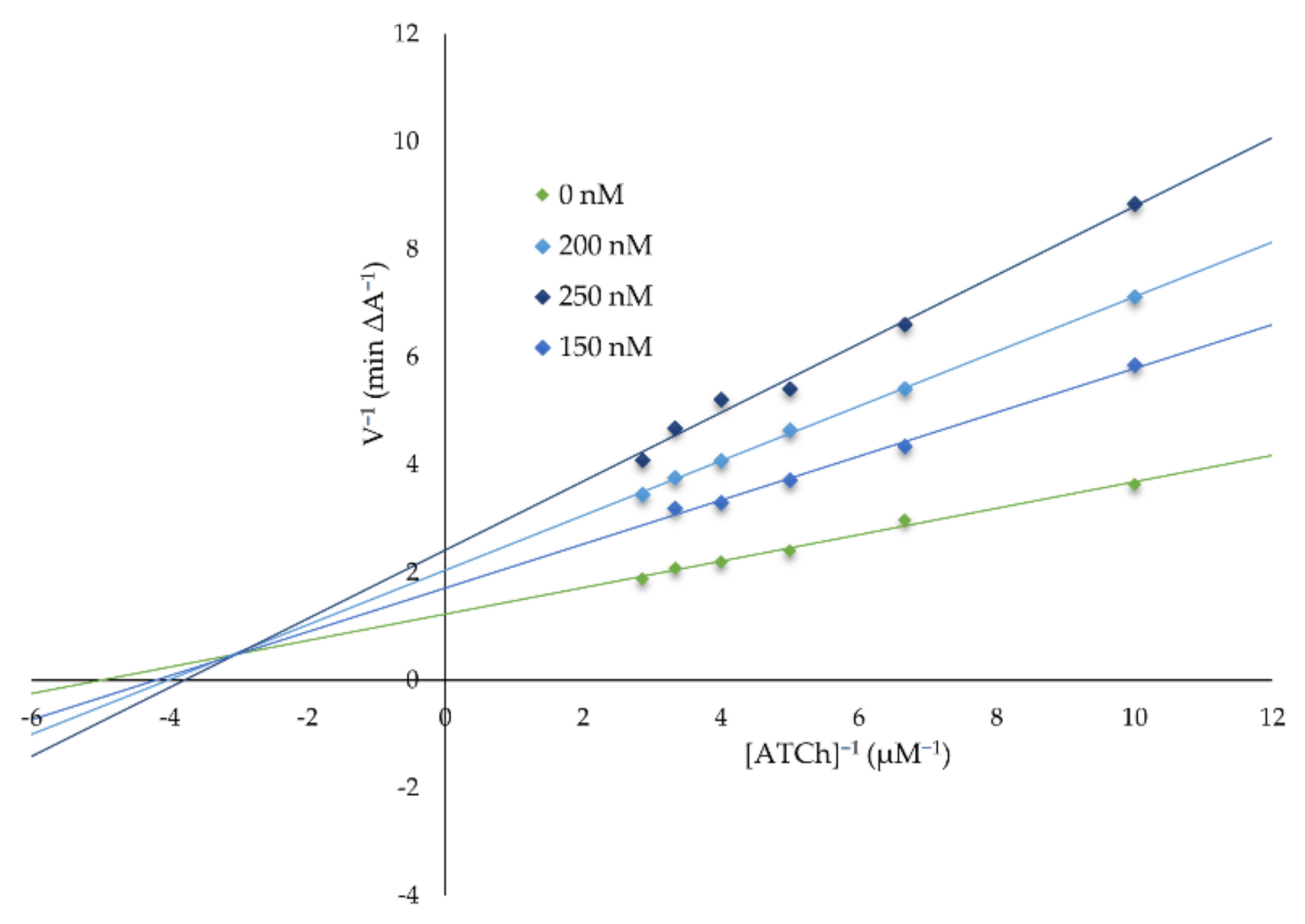
A Lineweaver-Burk plot presenting a mixed-type of BuChE inhibition by **3b**.

Kinetic studies were performed in order to reveal the mechanism of AChE inhibition by **3f** (Figure 1) and BuChE inhibition by **3b** (Figure 2) for the most potent compounds. Lineweaver–Burk plots were prepared to reveal the type of inhibition. The experiments were performed in triplicate. An analysis of the Lineweaver–Burk plots and K_m_ and V_max_ values showed increasing slopes and intercepts with an increasing inhibitor concentration, which suggested a mixed type of inhibition for both compounds.

#### 2.2.3. Antioxidant Activity Evaluation

Oxidative stress is regarded as a significant factor of pathogenesis in neurodegenerative disorders such as Alzheimer’s disease. The design of multifunctional drugs often involves the antioxidant properties of a drug candidate. The most potent compounds (**3b** and **3f**) have been evaluated as reactive oxygen species (ROS) scavengers using an ORAC-FL assay. Trolox was used a reference compound, and the results were compared to tacrine.

Both the **3b** and **3f** compounds demonstrated antioxidant activity against ROS significantly higher than that of tacrine (Table 1) in the ORAC-FL assay. The **3b** compound in a concentration range of (1–13 µM) revealed an approximately 35 higher antioxidant activity (TE = 0.4614 ± 0.056) than tacrine (TE = 0.0132 ± 0.009) in a concentration range of (1–10 µM). The **3f** compound with a longer alkyl chain revealed a slightly lower antioxidant activity (TE = 0.3254 ± 0.014) than **3b** in the same concentration range, but around 25 times higher than tacrine.

#### 2.2.4. In Vitro Hepatotoxicity Assay

The potential hepatotoxic activity of **3b** and **3f** at different concentrations were investigated on human hepatic stellate cells (HSCs). The hepatotoxicity of tacrine, due to its toxic metabolites, such as 7-hydroxytacrine, was the reason for its withdrawal. The compounds a concentration was chosen based on the IC_50_ results from the AChE inhibition test. The HSCs viability was determined using an MTT test. The results were presented as IC_50_ values, representing the inhibitory concentration which reduces the cell’s viability to 50% compared to the control (100% viability). The derivative **3b** with a shorter alkyl chain possessed a stronger hepatotoxic activity (IC_50_ = 31.96 ± 2.05 µM) than compound **3f** (IC_50_ = 49.75 ± 4.36 µM). Nevertheless, the IC_50_ values from the hepatotoxicity study are significantly higher than the IC_50_ results from the AChE and BuChE inhibition tests for those derivatives.

#### 2.2.5. Molecular Modeling

The tridimensional structure of the entrance and the interior of the binding pocket of AChE and BuChE enzymes, as well as the chemical character of their pocket-lining amino acids, determine the binding modes of the investigated ligands by enforcing conformational changes of a ligand upon interactions with the surrounding residues. (The main role of an aliphatic linker chain is to adapt the ligand’s geometry to the conditions inside the enzyme’s binding pocket, which depend directly on the amino acid context.

**Figure 3:**
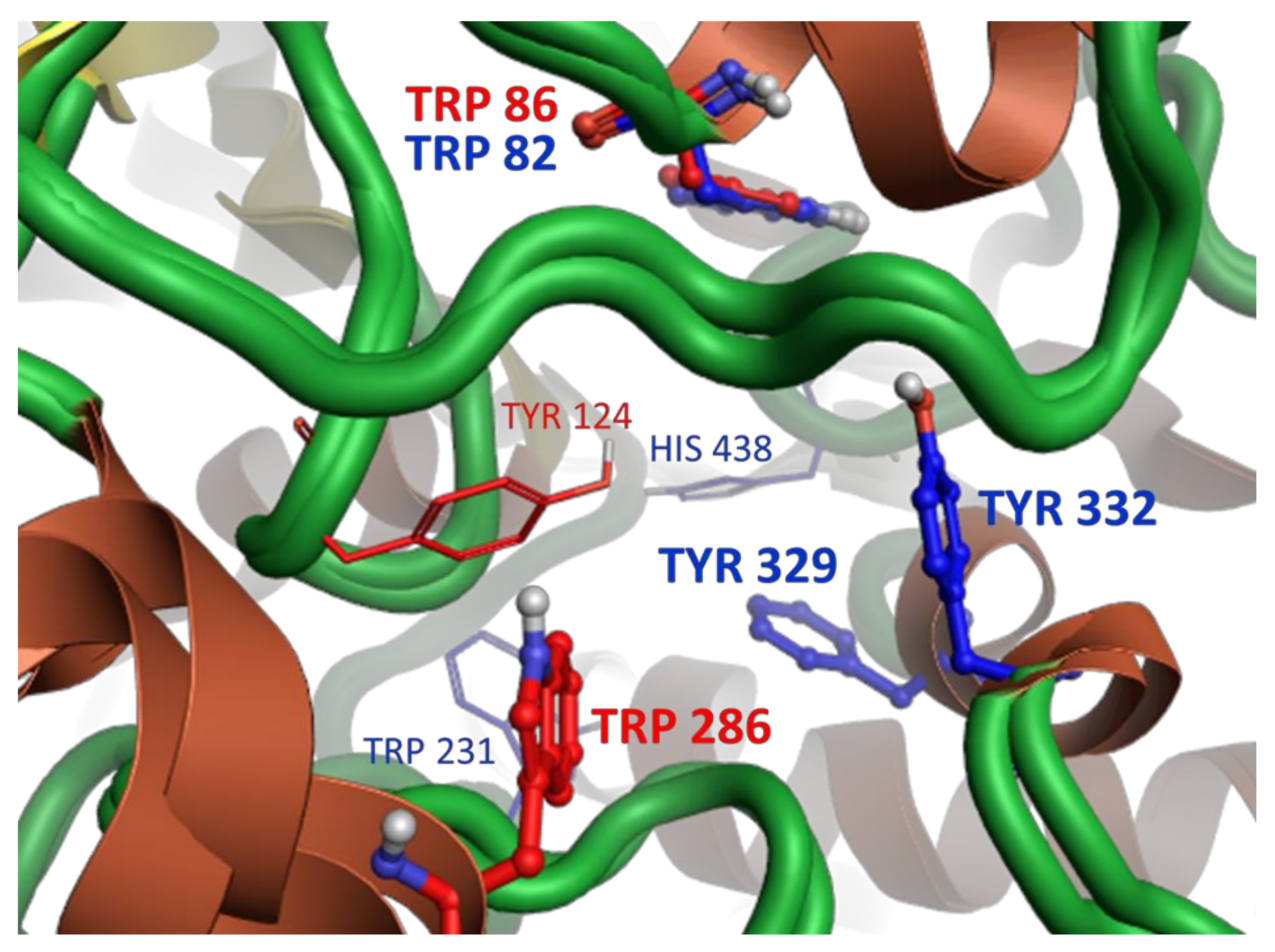
The cartoon model of the superimposed AChE’s and BuChE’s pockets with the ball-and-stick representations of the key ligand-binding aromatic residues in the pockets of AChE (red) and BuChE (blue). Note that both enzymes share the position of one ligand-binding region, which correspond to the TRP 86 and TRP 82 residues in AChE and BuChE, respectively.

The shape of the AChE’s pocket reminds one of a (~20 Å-long and ~8 Å-wide) tunnel, while BuChE’s pocket is more spherical with a diameter of about 15 Å. The abundance of aromatic amino acids is two times higher in AChE than in BuChE (especially in the catalytic anionic site, CAS). BuChE contains more hydrophilic and charged residues than AChE (particularly in the peripheral anionic site, PAS). The TRP 86 (in CAS) and TRP 286 (in PAS) are the key aromatic residues responsible for the interactions between the AChE’s binding pocket and the polyaromatic parts in the studied series of ligands (acridine and tacrine) (Figure 3). In the case of BuChE, this role is played by four aromatic residues: PHE 329, TYR 332, TRP 231, and TRP 82, which is separated from them by about ~10 Å, situated on the other side of CAS region (Figure 3).

**Figure 4:**
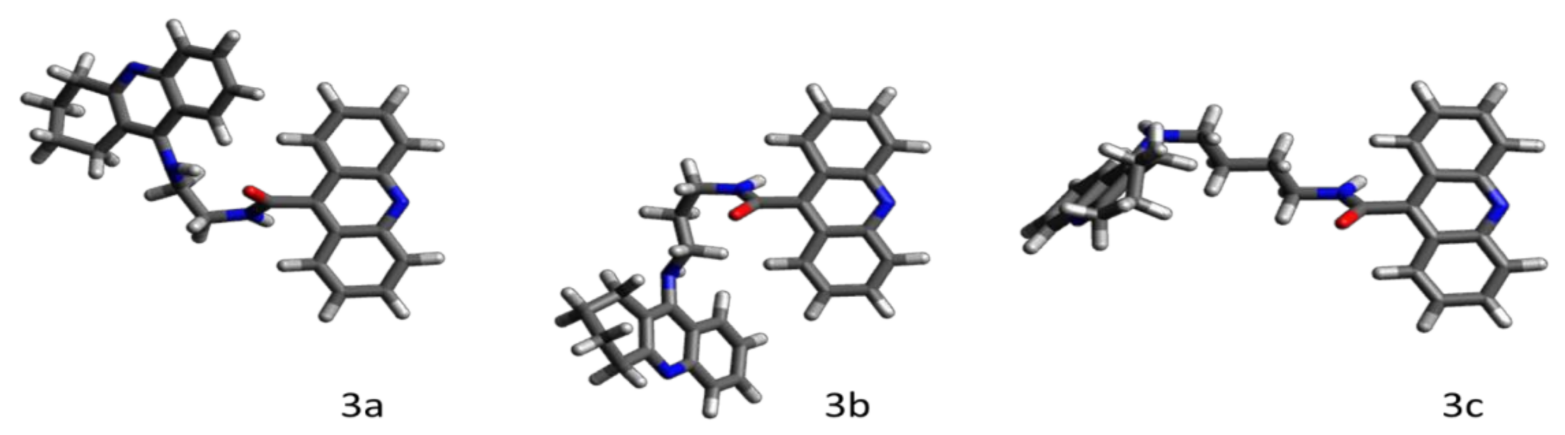
The thermally stable geometries of the smallest ligands in the studied series, calculated at the quantum-mechanical level of theory. Note that the bulky conformation of **3a** is additionally stabilized by the intramolecular NH…O hydrogen bond. Both **3b** and **3c** exhibit hook-shaped structures.

The quantum–chemical calculations of the studied series of ligands confirmed that the length of the polimethylene linker between the acridine and cyclopentaquinoline moieties correlates directly to the flexibility of the ligand. The linker chains with up to four methylene segments (**3a**–**3c**) are stiff due to steric and electronic effects resulting from a close proximity of the rigid polyaromatic cyclopentaquinoline and acridine moieties. For this reason, the smallest ligands favorably adopt particular geometries, which correspond to deep (i.e., separated by energy berries much higher the thermal fluctuations) local energetic minima (Figure 4). Note that once inside the binding pocket, those ligands may go through conformational changes induced by interactions with the surrounding amino acid residues.

The smallest and the most rigid ligand in the series, **3a**, in its thermally stable conformation (Figure 4, ligand **3a**), is too bulky to enter the ~8 Å-wide AChE’s pocket. In the unlikely event that **3a** adopted a less bulky (elongated) geometry and managed to enter the AChE’s pocket, its short and rigid linker chain would not allow the simultaneous parallel π-stacking interactions of both polyaromatic moieties with the key ligand-binding tryptophan residues (TRP 86 and TRP 286), which are separated by a distance of ~16 Å. The inability to sufficiently interact with both key binding residues at the same time (i.e., impossible dual binding mode) has a strong decreasing impact on **3a**’s binding affinity towards AChE, also manifested by the high value of its IC_50_.

The bulky **3a** passes through the wider entrance of the BuChE’s pocket and fits perfectly in its spacious interior, while reaching for the key ligand-binding aromatic amino acid residues TRP 231 (T-shaped π-stacking with acridine) and TRP 82 (parallel π-stacking with tacrine). For this reason, **3a** exhibits a considerable affinity towards BuChE, which is also manifested by a low value of its IC_50_.

As presented in Figure 4, ligands **3b** and **3c** exhibit similar hook-shaped structures, which can both pass through the entrances and fit well in the interiors of both enzymes’ pockets. **3b** and **3c** structurally unwind while fitting though the narrow AChE’s pocket, and both adopt very similar dual-binding modes, with acridine and tacrine parallelly π-stacked with TRP 86 and TRP 286, respectively. Both the binding poses of **3b** and **3c** are additionally stabilized by the hydrogen bonds between the hydroxy group of TYR 124 and the oxygen of the ligand’s amide group. The almost identical binding modes of **3b** and **3c** in the AChE’s pocket correspond to the similar high affinity of those ligands, also confirmed by the similar values of their IC_50_.

Both **3b** and **3c** remain hook-shaped after entering the wider BuChE’s pocket and adopt similar dual binding modes, with tacrine parallelly π-stacked with TRP 82, and acridine involved in the T-shaped π-stacking interactions with TRP 231 and PHE 329. A slightly wider spacing between those aromatic residues and the acridine moiety of **3c** is responsible for its lower affinity towards BuChE with respect to **3b**, reflected in the three-time difference in their IC_50_ values.

For those longer than the tetramethylene linker chains (**3d**–**3g**), we observed an unrestrained rotational motion between the methylene segments resulting in—proportional to the linker’s length—an increased flexibility of the aliphatic chains of those ligands.

**Figure 5:**
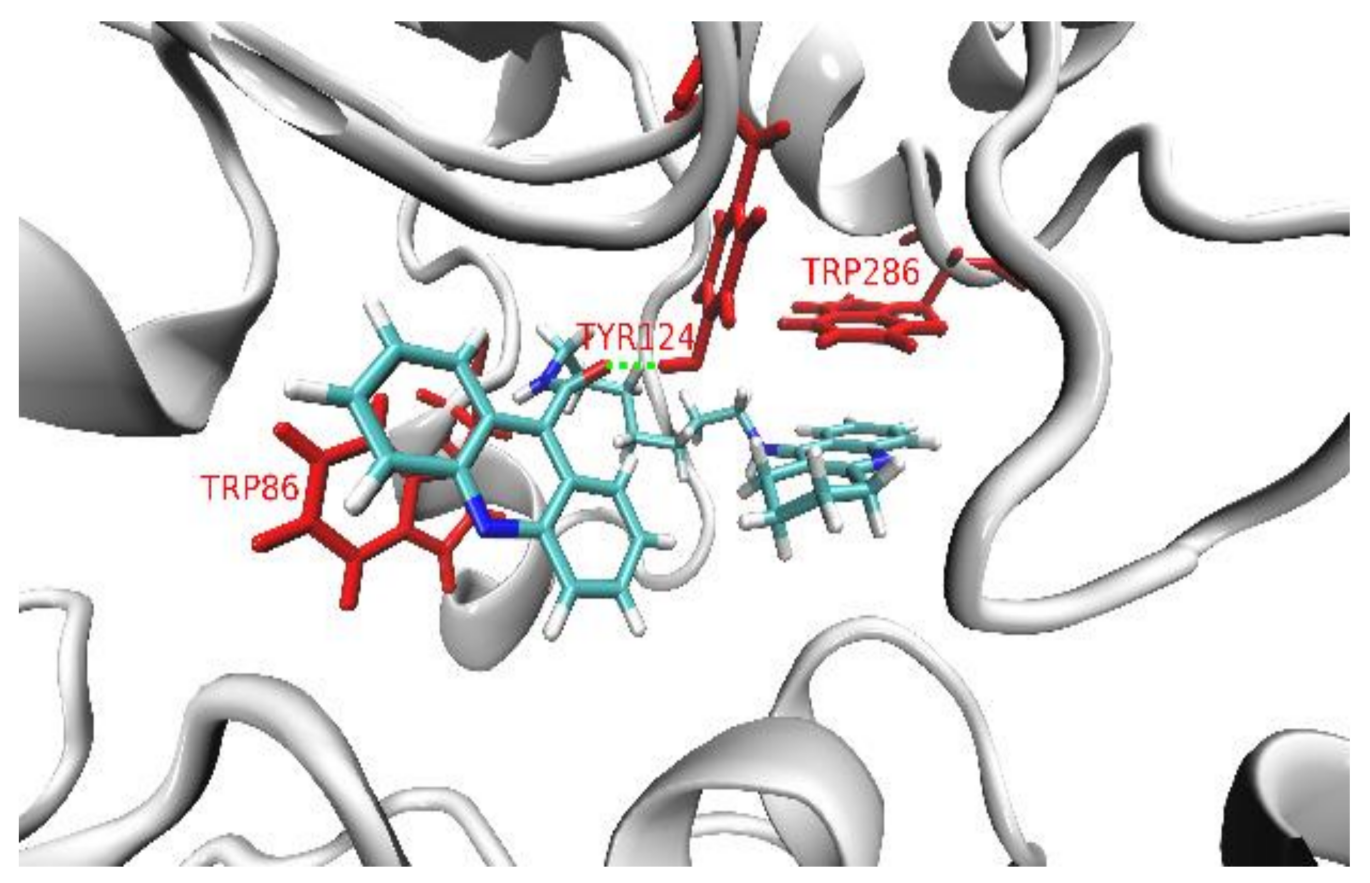
The binding mode of the **3f** ligand inside the pocket of AChE. The key ligand-binding aromatic residues in the pocket of AChE are marked in red.

The **3d**–**3h**, thanks to their flexible linker chains, most likely enter the narrow tunnel of the AChE’s pocket in a worm-like manner with acridine acting as the head part. While acridine reaches for the TRP 86 residue deep inside the pocket, the cyclopentaquinoline goes straight down the line towards TRP 286 (Figure 5). In order to fit though, the flexible linkers of the **3d**–**3h** ligands unwind inside the narrow AChE’s pocket. The main role of the flexible linker chain is to conformationally rearrange ligands inside the enzyme’s binding pocket, and help both polyaromatic parts of the ligand (acridine and cyclopentaquinoline) efficiently π-stack with the AChE’s key tryptophan residues. This is the case for **3d**, whose unwound pentylomethylene linker chain has a perfect length that allows both acridine and cyclopentaquinoline to closely π-stack with TRP 86 and TRP 286, respectively. That strong dual binding mode, additionally stabilized by the hydrogen bond between the hydroxy group of TYR 124 and the oxygen of the ligand’s amide group, corresponds to a very high affinity of **3d** towards AChE confirmed by its very low value of IC_50_. We observed—almost identical to **3d**’s binding modes of the polyaromatic parts of the **3f** and **3h** ligands—that though their >16 Å (i.e., longer than TRP 86—TRP 286 distance), unwound linkers had to rearrange geometries inside the crowded AChE’s binding pocket in order to steer the polyaromatic moieties towards efficient π-stacking interactions with the key tryptophan ligands binding-residues, additionally stabilized by the hydrogen bonds between the hydroxy group of TYR 124 and the oxygen of the ligands’ amide groups. Importantly, the odd number of the methylene segments in the linker chain corresponds to a reciprocal conformation of acridine and cyclopentaquinoline that favors their suitable overlap with the TRP 86 and TRP 286 rings, and in turn promotes strong π-stacking interactions between those residues and both polyaromatic moieties. For this reason, **3d**, **3f**, and **3h** all exhibit a high affinity towards AChE. On the other hand, possessing an even number of the methylene segments in the linker chain corresponds to a reciprocal conformation of acridine and cyclopentaquinoline, which is disadvantageous for a suitable overlapping of the stacking rings in a dual binding mode, which is therefore responsible for a drastic drop in the affinity towards AChE of both **3e** and **3g**.

Considering the architecture of the BuChE’s binding pocket with the two main ligand-binding regions separated by only ~10 Å within CAS, in order to achieve dual binding modes, the structures of **3d**–**3h** ligands must adopt compact poses with rather collapsed linker chains; in doing so, they would allow both acridine and tacrine π-stacking close to each other key aromatic residues. The wide entrance, as well as the spacious interior, enable the bulky **3d**–**3h** ligands with collapsed linker chains to enter the BuChE’s pocket. Such compact poses can be additionally stabilized by the intramolecular NH…O hydrogen bond (**3d** and **3g**), or by π-stacking interactions between acridine and tacrine moieties (**3h**).

**Figure 6:**
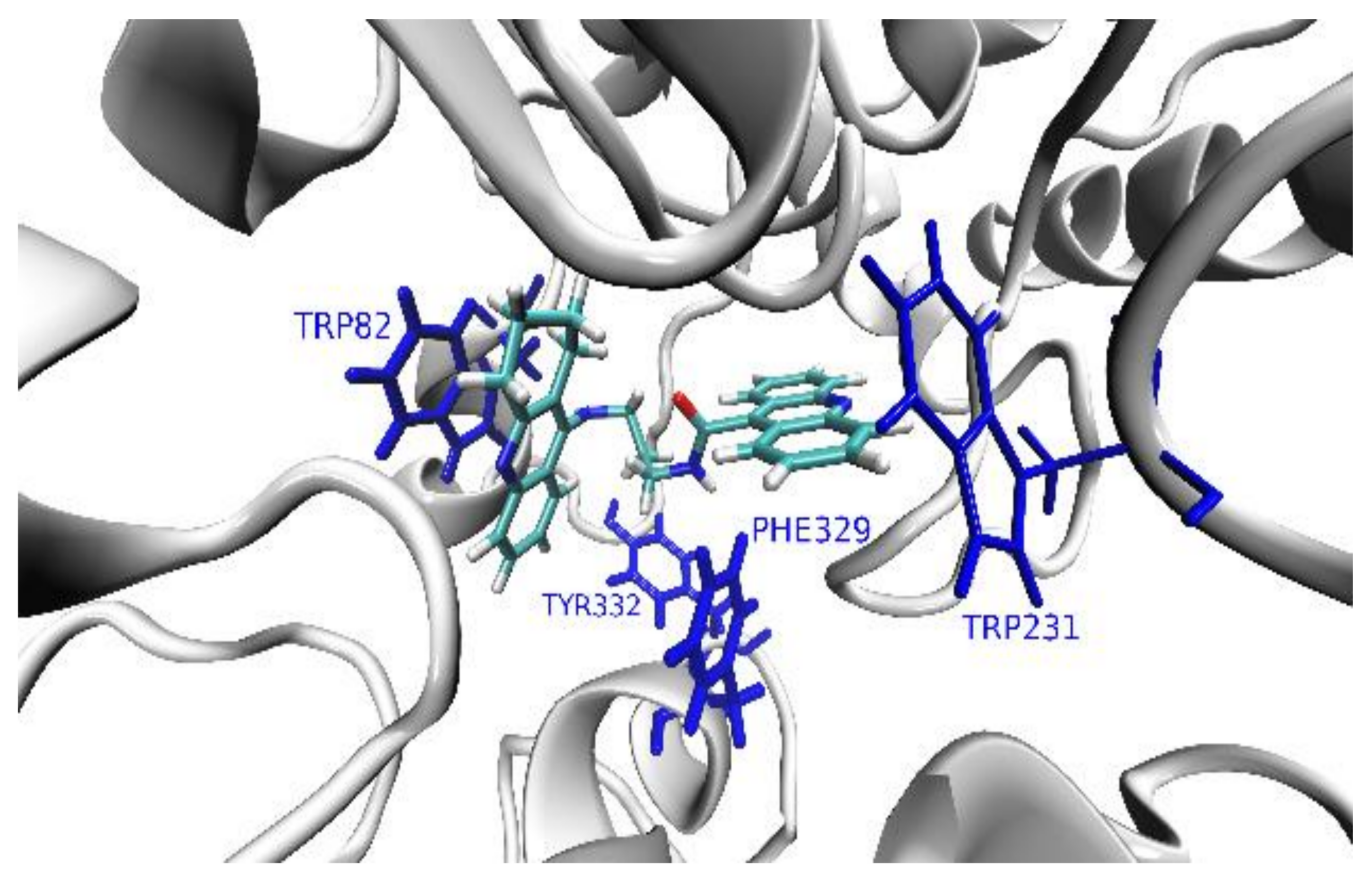
The binding mode of the **3b** ligand inside the pocket of BuChE. The key ligand-binding aromatic residues in the pocket of BuChE are marked in blue.

Although **3d** adopts a hook-shaped geometry inside the BuChE’s pocket, it is bound in the opposite way to **3b** and **3c**, with its acridine binding TRP 82 residue via a parallel π-stacking interaction. At the same time, the cyclopentaquinoline moiety creates a close T-shaped π-stacking interaction with PHE 329 residue (Figure 6). Such a sterically favorable geometry—additionally stabilized by the intramolecular hydrogen bond—allows **3d** to obtain a stronger interaction with the TRP 82 residue via acridine instead of a bulkier cyclopentaquinoline moiety, which is responsible for the several-time increase of the affinity of **3d** towards BuChE with respect to both **3b** and **3c**.

Although **3e** also adopts a hook-shaped geometry with the acridine moiety involved in a weaker (parallel displaced) π-stacking interaction with TRP 82, its additional methylene segment pushes the tacrine moiety away from the other ligand-binding region of the pocket, which contains the PHE 329, TYR 332, and TRP 231 residues. The singular binding mode is responsible for the very low affinity of **3e** towards BuChE, which is observed as a very high value of IC_50_.

The heptamethylene linker chain is long enough to adopt tangled structures and therefore to successfully rearrange the reciprocal positions of the polyaromatic moieties of **3f** with respect to the key ligand-binding residues. Besides the parallel π-stacking interaction between acridine and TRP 82, the cyclopentaquinoline moiety is involved in two T-shaped π-stacking interaction with PHE 329 and TRP 231 residues. The acridine moiety of **3g**, in addition to the close π-stacking with TRP 82, interacts with the HIS 438 residue, while the cyclopentaquinoline moiety binds via a T-shaped π-stacking interaction with the PHE 329 residue. Such a dual binding mode, together with the additional stabilization of the ligand’s geometry by the intramolecular hydrogen bond, contributes to the slightly higher affinity of **3g** than **3f** towards BuChE, and in turn, the slightly lower value of **3g**’s IC_50_.

Although the non-amethylene linker chain of **3h** collapses to such an extent that the polyaromatic moieties can interact intermolecularly via π-stacking interactions, its dual binding mode is sterically restricted to a weaker displaced parallel π-stacking interaction between acridine and TRP 82, and the T-shaped π-stacking interaction with PHE 329. Moreover, the geometry of the collapsed **3h** is much bulkier than the hook-shaped conformations of the smaller (**3a**–**3g**) ligands. The hook-like structures can most probably enter the BuChE’s pocket more easily. The weak dual binding mode is less able to enter the BuChE’s pocket conformation, which results in its lower affinity as indicated by relatively high IC_50_.

#### 2.2.6. ADMET Analysis

The pharmacokinetic profiles of compounds are very varied. The physicochemical profiles of **3a**, **3b**, and **3c** indicate that these structures are suitable candidates for the drug structure and fulfilment of the Lipinski rule [31], Egan rule [32], and Veber rule [33]. The **3d** and **3e** compounds fulfilled the Veber rule only. The basic physicochemical properties of all compounds are summarized in Table 2.

AdmetSAR (version 2.0) software (admetSAR 2019) indicated optimal human plasma protein binding for compounds **3a**, **3b**, and **3c** (86.87%, 90.29%, 91.70% respectively). In addition, the ACD/Percepta (version 14.0.0) software (Advanced Chemistry Development, Inc., Metropolitan Toronto, ON, Canada) showed a high distribution volume (>11 L/kg) for all compounds in the series. Gastrointestinal absorption capacity was confirmed for compounds **3a**, **3b**, **3c**, **3d**, and **3e** (admetSAR 2.0, SwissADME). The blood–brain barrier permeation prediction results (ACD/Percepta, admetSAR 2.0) indicated sufficient brain penetration for all structures, but significantly better results were obtained for **3a**, **3b**, and **3c**.

The ProTox II analysis classified **3a** into toxicity class five (predicted LD_50_ 2100 mg/kg), **3b** into toxicity class three (predicted LD_50_ 200 mg/kg), and all remaining structures into toxicity class four (predicted LD_50_ 1000 mg/kg). Compound **3a** had very promising results for the detailed prediction of its toxicity profile, namely a 0.5 probability of mutagenicity (1 weak positive toxicity prediction test result of 17 different predictions). **3b** and **3c** showed a 0.64 and 0.58 probability of immunotoxicity, respectively, and a 0.55 and 0.53 probability of mutagenicity, respectively (2-weak positive toxicity prediction test results of 17 different predictions).

## 3. Discussion

AD is a devastating, neurodegenerative disorder affecting the CNS, mostly in people above 65 years. The pathogenesis of AD has a multifactorial and complicated nature [2]. As a consequence, the precise mechanism of the development of AD has not been fully revealed. Many pathological hallmarks and processes have been proposed as the cause of the disease [4]. Among them, the impairment of cholinergic neurotransmission was widely studied and generally approved [9]. In the center of the cholinergic hypothesis are cholinesterases. Increased levels of AChE and BuChE lead to the reduction of the ACh level in the synaptic cleft [9]. The decreased level of ACh results in cognitive impairment and neuroinflammation [11]. Moreover, the disruption in cholinergic neurotransmission is related to the Aβ by promoting the formation of highly neurotoxic complexes between the AChE and Aβ [12,13]. Although the cholinergic hypothesis has lost its relevance recently, new studies bring important insights into the interplay of cholinergic neurotransmission disruption and Aβ [13].

A series of cyclopentaquinoline and 9-acridinecarboxylic acid derivatives were designed, synthesized, and biologically evaluated as potential AD drug candidates. We used cyclopentaquinoline as an analog of tetrahydroacridine to minimalize the toxic effects and simultaneously maintain the high biological activity of the novel derivatives [24]. Our investigation of the novel derivatives included the inhibition potency against AChE and BuChE, kinetic studies, and an evaluation of antioxidant activity and toxicity. The inhibition studies against AChE and BuChE were performed using a modified version of Ellman’s method [29,34].

All compounds exhibited satisfactory inhibitory activity in the nanomolar range. The series of compounds has a higher inhibitory activity and selectivity for BuChE. These results are important when taking into consideration the fact that, during the development of Alzheimer’s disease, BuChE becomes more active than AChE [35]. The most potent compound against AChE is **3f** (IC_50_ = 113.34 nM), which is approximately two times higher than tacrine (IC_50_ = 226.97 nM) and four times more potent than bistacrine (IC_50_ = 405.10 nM). The **3f** compound represents a derivative with a longer alkyl chain consisting of seven methylene groups. It also represents satisfactory activity against BuChE (IC_50_ = 203.52 nM). The length and flexibility of the heptamethylene linker chain in **3f** provides successful rearranging of the reciprocal position of the polyaromatic moieties of acridine and cyclopentaquinoline with respect to the crucial ligand-binding amino acid residues. With regards to BuChE, the most potent compound was **3b** (IC_50_ = 103.73 nM) with a shorter alkyl chain consisting of three methylene groups. The **3b** derivative was two times more potent than bistacrine (IC_50_ = 226 nM) and revealed satisfactory activity against AChE (IC_50_ = 272.33 nM). Derivative **3b** easily enters to pockets of both enzymes. A hook-shaped structure allows it to pass the narrow entrance of the enzymes and interact with the key binding amino acid residues. Compound **3c** exhibited satisfactory IC_50_ values (around 200 nM) for both enzymes. For AChE, in particular, the IC_50_ values for **3c** and **3b** are almost identical. This similarity is due to the hook-shaped structure and dual binding modes with acridine and cyclopentaquinoline parallel pi-stacked characteristic for **3b** and **3c** revealed in molecular modeling studies. Derivative **3a**, with and ethylene alkyl chain combining a cyclopentaquinoline ring with a 9-acridinecarboxylic moiety, exhibited the highest IC_50_ values for both enzymes. The **3a** derivative, due to its bulky structure, cannot enter into AChE’s narrow pocket, which is reflected in very high IC_50_ values for AChE. The wider entrance to BuChE’s pocket allows it to reach key ligand binding aromatic residues in the CAS of BuChE. Consequently, the selectivity **3a** for BuChE is high.

According to the results, the **3b** and **3f** derivatives as promising dual AChE and BuChE inhibitors were chosen for further analysis. Kinetic studies revealed a mixed-type of inhibition for both the **3b** and **3f** derivative. In Lineweaver–Burk plots, the interceptions of the lines occur above the *x*-axis at the same point. Moreover, an analysis of plots showed increasing slopes (increased K_m_ values) and intercepts (decreased V_max_ values) with an increasing inhibitor concentration, which confirmed a mixed-type of inhibition [36].

Hepatotoxicity was the reason of the withdrawal of the first drug of Alzheimer’s disease, tacrine. The mechanism of tacrine hepatotoxicity has not been fully elucidated. It is believed that the unbound amino group located at position nine in the tacrine rings plays a role in the hepatotoxicity mechanism. Tacrine is metabolized by CYP1A2 [37]. Several studies have revealed a possible mechanism related to toxic metabolites, such as 7-hydroxytacrine. Recently, metabolomic studies revealed that tacrine directly disrupts the proton gradient of mitochondria and decreases ATP production, which then induces oxidative stress and mitochondrial dysfunction [37]. Therapy with tacrine has been associated with a significant elevation of liver enzymes, which in some cases can lead to jaundice and liver injury [38]. The current pharmacotherapy of Alzheimer’s disease based on cholinesterase inhibitors (donepezil, rivastigmine, and galantamine) and memantine is well tolerated by patients and safely used in terms of hepatotoxicity risk. However, liver function patients with dementia should be monitored due to difficulties in identifying symptoms and in communication with dementia patients [39]. Nevertheless, tacrine is not currently used in the pharmacotherapy of Alzheimer’s disease, and designing drugs with a structural similarity to tacrine requires hepatotoxicity studies. An hepatotoxicity study on HSCs line revealed a higher toxic activity for the **3b** derivative (IC_50_ = 31.96 ± 2.05 µM) with a shorter alkyl linker than compound **3f** (IC_50_ = 49.75 ± 4.36 µM). However, the concentrations leading to a 50% reduction of the cells viability were significantly higher than results from the cholinesterase inhibition study.

Antioxidant properties in the drug design for Alzheimer’s disease have become desirable due to the oxidative stress underlying the pathology and progression of this devastating disease [40]. The most potent compounds have been evaluated as ROS scavengers using a fluorometric ORAC-FL method. Both **3b** and **3f** derivatives showed significantly higher antioxidant activity than tacrine (35 and 25 times higher respectively).

The ADMET analysis revealed the superiority of the **3b** derivative. It fulfills Lipinski’s rule of five, Egan’s rule, and Veber’s rule. The **3b** compound binds in 90.29% with the human plasma protein and possesses suitable brain penetration. It was classified into toxicity class 3.

The molecular modeling study indicated that the inhibitory activity of the investigated series of ligands towards AChE and BuChE enzymes depends on:

1. the amino acid context and, in turn, the tridimensional structures of the entrance and the interior of the binding pocket of an enzyme,
2. the reciprocal conformational of the acridine and cyclopentaquinoline moieties depending on the (odd or even) number of the methylene segments in the aliphatic linker chain,
3. the presence of extra- and intramolecular hydrogen bonds, which additionally stabilize both the ligand’s conformation and binding pose inside the pocket,
4. the length and flexibility of the linker chain, with longer conformational rearrangements of aliphatic chains promoting more favorable positions of ligands inside the pocket.

Molecular modeling studies of enzyme docking confirmed the results obtained in in vitro enzymatic studies. Simultaneously, we explained the mechanism of interaction of individual pharmacophore groups with the binding site. This will allow for further research in this area and will be used for the development and design of the new cholinesterase inhibitors.

Nowadays, cholinesterases inhibitors are the basis of the pharmacotherapy of Alzheimer’s disease. Their use in patients with Alzheimer’s disease is associated with specific side effects, both somatic (nausea, vomiting, diarrhea, headache, and weight loss) and psychiatric (depression, insomnia, and sleep architecture disruptions) resulting from the increased level of ACh [41]. However, it is difficult to assess the risk of psychiatric side effects developing because of the influence of the neurodegenerative process on their development [41,42]. The application of strong cholinesterases inhibitors in small doses or special formulations may result in increased effectiveness of therapy and side effects reduction.

## 4. Materials and Methods

### 4.1. Synthesis

The binding modes of the ligands inside the pocket (Figures S1–S14).

The chemical reagents used in the synthesis were purchased from commercial sources. All chemical reactions were monitored by thin-layer chromatography (TLC) and a UV lamp. The organic solvents were removed using rotary evaporation under reduced pressure. Flash chromatography (column 15 µm SiHP, 12 g, 4 g, Interchim, Montluçon, France) was used to purify obtained compounds. The infrared (IR) spectra were recorded by a Mattson Infinity Series Fourier transform infrared (FT-IR) spectrophotometer in ATR (Nicolet 6700, Thermo Scientific, Waltham, MA, USA). ^1^H NMR and ^13^C NMR spectra were recorded with a Bruker Advance III 600 MHz spectrometer with tetramethylsilane (TMS) as an internal standard. The elemental analysis, including mass spectra and high-resolution mass spectra (HRMS), were performed using an Agilent Accurate-Mass Q-TOF LC/MS G6520B system with a dual electrospray (DESI) source (Agilent Technologies, Santa Clara, CA, USA). The detector in the analysis was tuned in a positive mode with the use of an Agilent ESI-L tuning mix in high-resolution mode (4 GHz). The determination of the melting point (mp) for each compound was performed on an electrothermal apparatus in open capillaries and was uncorrected. Scheme 1 presents the two-step synthesis of the novel compounds.

#### 4.1.1. General Procedure of Compound **2a**–**2h** Synthesis

9-acridinecarboxylic acid to the 10 mL of tetrahydrofuran (THF) and stirred in an ice bath for 2 h. Then, N-methylmorpholine and an acid activator 2-chloro-4,6-dimethoxy-1,3,5-triazine (CDMT) were added to the mixture and stirred in an ice bath for the next 4 h. In the end, dissolved THF (3 mL) diamine **1a**–**1h** was quickly added and reaction was continued at room temperature with constant stirring for 24 h. A physical and elemental analysis was performed for each compound.

##### N-[2-({1H,2H,3H-cyclopenta[b]quinolin-9-yl}amino)ethyl]acridine-9-carboxamide (**2a**)

9-acridinecarboxylic acid (0.168 g, 0.75 mmol) was added to 10 mL THF and dissolved. The mixture was stirred in an ice bath for 2 h. Next, CDMT (0.132 g, 0.75 mmol) and N-methylmorpholine (0.083 mL, 0.75 mmol) were added to the mixture and stirred in an ice bath for another 4 h. Then, once dissolved in 3 mL of THF, compound **1a** (0.171 g, 0.75 mmol) was quickly added to the mixture and stirred continuously for 24 h.

Compound **2a**: greenish solid (yield: 26,28%); mp 130–132 °C; FTIR-ATR ν (cm^−1^): 752.8; 1279.1; 1367.4; 1463.1; 1569.4; 2942.2; 3195.1; ^1^H NMR (600 MHz, Methanol-*d*_4_) δ 8.41 (d, *J* = 8.6 Hz, 1H, ArH), 8.18–8.21 (m, 2H, ArH), 7.94–7.97 (m, 2H, ArH), 7.83–7.89 (m, 4H, ArH), 7.65 (t, *J* = 8.4 Hz, 1H, ArH), 7.45–7.49 (m, 2H, ArH), 4.28 (t, *J* = 6.0 Hz, 2H, CH_2_), 4.06 (t, *J* = 6.0 Hz, 2H, CH_2_), 3.55–3.51 (m, 2H, CH_2_), 3.20 (t, *J* = 7.9 Hz, 2H, CH_2_), 2.29 (p, *J* = 7.8 Hz, 2H, CH_2_); ^13^C NMR (151 MHz, MeOD) δ 175.7, 162.1, 152.8, 138.7, 132.1, 130.8, 128.3, 126.7, 126.1, 124.9, 122.4, 122.1, 117.3, 112.7, 48.2, 39.9, 31.4, 31.2, 20.7; MS (ESI) *m*/*z*: 85.1, 185.1, 206.0, 249.1; MS-HR (ESI) calcd for C_28_H_24_N_4_O: 432.1963, found: 432.1950.

##### N-[3-({1H,2H,3H-cyclopenta[b]quinolin-9-yl}amino)propyl]acridine-9-carboxamide (**2b**)

9-acridinecarboxylic acid (0.155 g, 0.69 mmol) was added to 10 mL THF and dissolved. The mixture was stirred in an ice bath for 2 h. Next, CDMT (0.122 g, 0.69 mmol) and N-methylmorpholine (0.076 mL, 0.69 mmol) was added to the mixture and stirred in an ice bath for another 4 h. Then, once dissolved in 3 mL of THF, compound **1b** (0.167 g, 0.69 mmol) was quickly added to the mixture and stirred continuously for 24 h.

Compound **2b**: yellow-green solid (yield: 26.28%); mp 138–141 °C; FTIR-ATR ν (cm^−1^): 750.8; 1275.1; 1373.8; 1457.8; 1558.5; 2917.2; 3197.0; ^1^H NMR (600 MHz, Methanol-*d*_4_) δ 8.37 (d, *J* = 8.5 Hz, 1H, ArH), 8.24 (dd, *J* = 8.8 Hz, 2H, ArH), 8.12 (dd, *J* = 8.5 Hz, 2H, ArH), 7.91–7.94 (m, 2H, ArH), 7.76–7.83 (m, 2H, ArH), 7.66–7.70 (m, 2H, ArH), 7.61 (t, *J* = 8.3 Hz, 1H, ArH), 4.06 (t, *J* = 7.0 Hz, 2H, CH_2_), 3.84 (t, *J* = 6.8 Hz, 2H, CH_2_), 3.42–3.46 (m, 2H, CH_2_), 3.17 (t, *J* = 7.9 Hz, 2H, CH_2_), 2.25 (dp, *J* = 20.8, 7.3 Hz, 4H, CH_2_); ^13^C NMR (151 MHz, MeOD) δ 168.2, 148.2, 142.1, 130.9, 128.3, 126.9, 125.3, 125.1, 122.2, 122.1, 121.8, 117.9, 112.6, 42.3, 36.9, 32.2, 31.1, 30.6, 22.0; MS (ESI) *m*/*z*: 149.0, 185.1, 223.1, 263.1; MS-HR (ESI) calcd for C_29_H_26_N_4_O: 446.2119, found: 446.2106.

##### N-[4-({1H,2H,3H-cyclopenta[b]quinolin-9-yl}amino)butyl]acridine-9-carboxamide (**2c**)

9-acridinecarboxylic acid (0.114 g, 0.51 mmol) was added to 10 mL THF and dissolved. The mixture was stirred in an ice bath for 2 h. Next, CDMT (0.089 g, 0.51 mmol) and N-methylmorpholine (0.056 mL, 0.51 mmol) was added to the mixture and stirred in an ice bath for another 4 h. Then, once dissolved in 3 mL of THF, compound **1c** (0.13 g, 0.51 mmol) was quickly added to the mixture and stirred continuously for 24 h.

Compound **2c**: yellow solid (yield: 25.77%); mp 140–143 °C; FTIR-ATR ν (cm^−1^): 756.8; 1258.3; 1367.5; 1458.0; 1541.1; 2915.6; 3233.4; ^1^H NMR (600 MHz, Methanol-*d*_4_) δ 8.37 (d, *J* = 8.4 Hz, 1H, ArH), 8.23 (dd, *J* = 8.8 Hz, 2H, ArH), 8.07 (dd, *J* = 8.6 Hz, 2H, ArH), 7.85–7.94 (m, 3H, ArH), 7.78 (d, *J* = 8.4 Hz, 1H, ArH), 7.59–7.68 (m, 3H, ArH), 3.96 (t, *J* = 6.9 Hz, 2H, CH_2_), 3.75 (t, *J* = 6.7 Hz, 2H, CH_2_), 3.41–3.46 (m, 2H, CH_2_), 3.16 (t, *J* = 7.9 Hz, 2H, CH_2_), 2.24 (p, *J* = 7.7 Hz, 2H, CH_2_), 1.92–1.97 (m, 4H, CH_2_); ^13^C NMR (151 MHz, DMSO) δ 168.3, 166.4, 148.6, 148.3, 146.9, 142.9, 131.1, 129.7, 128.7, 128.4, 127.2, 126.0, 123.6, 122.2, 122.1, 119.4, 112.5, 44.2, 40.3, 34.6, 31.1, 29.1, 26.8, 23.2; MS (ESI) *m*/*z*: 185.1, 206.0, 239.2, 282.2; MS-HR (ESI) calcd for C_30_H_28_N_4_O: 460.2293, found: 460.2263.

##### N-[5-({1H,2H,3H-cyclopenta[b]quinolin-9-yl}amino)pentyl]acridine-9-carboxamide (**2d**)

9-acridinecarboxylic acid (0.163 g, 0.73 mmol) was added to 10 mL THF and dissolved. The mixture was stirred in an ice bath for 2 h. Next, CDMT (0.128 g, 0.73 mmol) and N-methylmorpholine (0.08 mL, 0.73 mmol) was added to the mixture and stirred in an ice bath for another 4 h. Then, once dissolved in 3 mL of THF, compound **1d** (0.197 g, 0.73 mmol) was quickly added to the mixture and stirred continuously for 24 h.

Compound **2d**: light yellow solid (yield: 21.84%); mp 168–172 °C; FTIR-ATR ν (cm^−1^): 761.8; 1273.9; 1371.8; 1466.5; 1558.0; 2925.8; 3209.0; ^1^H NMR (600 MHz, Methanol-*d*_4_) δ 8.30 (d, *J* = 8.2 Hz, 1H, ArH), 8.22 (dd, *J* = 8.8 Hz, 2H, ArH), 8.08 (dd, *J* = 8.4 Hz, 2H, ArH), 7.87–7.94 (m, 1H, ArH), 7.80 (t, *J* = 8.2 Hz, 1H, ArH), 7.72–7.75 (m, 1H, ArH), 7.62–7.66 (m, 1H, ArH), 7.55 (t, *J* = 8.4 Hz, 1H, ArH), 3.89 (t, *J* = 7.3 Hz, 2H, CH_2_), 3.70 (t, *J* = 7.0 Hz, 2H, CH_2_), 3.40–3.46 (m, 2H, CH_2_), 3.16 (t, *J* = 7.9 Hz, 2H, CH_2_), 2.28 (p, *J* = 7.8 Hz, 2H, CH_2_), 1.85–1.95 (m, 4H, CH_2_), 1.70 (p, *J* = 7.8, 7.3 Hz, 2H, CH_2_); ^13^C NMR (151 MHz, MeOD) δ 176.8, 161.7, 152.6, 142.5, 138.7, 131.9, 130.9, 128.3, 126.7, 125.7, 125.1, 122.2, 122.2, 112.1, 44.4, 39.4, 31.4, 31.2, 30.6, 28.8, 22.4, 21.4; MS (ESI) *m*/*z*: 185.1, 206.0, 253.2, 296.2, 447.2; MS-HR (ESI) calcd for C_31_H_30_N_4_O: 474.2454, found: 474.2419.

##### N-[6-({1H,2H,3H-cyclopenta[b]quinolin-9-yl}amino)hexyl]acridine-9-carboxamide (**2e**)

9-acridinecarboxylic acid (0.173 g, 0.77 mmol) was added to 10 mL THF and dissolved. The mixture was stirred in an ice bath for 2 h. Next, CDMT (0.136 g, 0.77 mmol) and N-methylmorpholine (0.085 mL, 0.77 mmol) was added to the mixture and stirred in an ice bath for another 4 h. Then, once dissolved in 3 mL of THF, compound **1e** (0.22 g, 0.77 mmol) was quickly added to the mixture and stirred continuously for 24 h.

Compound **2e:** orange oil (yield: 10.39%); FTIR-ATR ν (cm^−1^): 757.0; 1255.6; 1364.2; 1458.4; 1562.1; 2916.8; 3253.1; ^1^H NMR (600 MHz, Methanol-*d*_4_) δ 8.22 (dd, *J* = 8.8 Hz, 2H, ArH), 8.14 (d, *J* = 8.6 Hz, ArH), 8.08 (dd, *J* = 8.6 Hz, 2H, ArH), 7.88–7.93 (m, 2H, ArH), 7.75 (d, *J* = 8.3 Hz, 1H, ArH), 7.60–7.66 (m, 3H, ArH), 7.44 (t, *J* = 7.3 Hz, 1H, ArH), 3.71–3.76 (m, 2H, CH_2_), 3.67 (t, *J* = 7.0 Hz, 2H, CH_2_), 3.30 (t, *J* = 7.2 Hz, 2H, CH_2_), 3.02 (t, *J* = 7.8 Hz, 2H, CH_2_), 2.16 (p, *J* = 7.6 Hz, 2H, CH_2_), 1.80 (dp, *J* = 21.1, 7.1 Hz, 4H, CH_2_), 1.58–1.67 (m, 2H, CH_2_), 1.33–1.41 (m, 2H, CH_2_); ^13^C NMR (151 MHz, MeOD) δ 167.7, 163.8, 148.2, 142.5, 130.9, 130.6, 128.2, 126.7, 125.1, 125.0, 122.4, 122.3, 121.7, 117.9, 112.2, 53.7, 39.4, 32.3, 31.0, 30.9, 28.9, 26.5, 18.4; MS (ESI) *m*/*z*: 185.1, 206.0, 310.2; MS-HR (ESI) calcd for C_32_H_32_N_4_O: 488.2597, found: 488.2576.

##### N-[7-({1H,2H,3H-cyclopenta[b]quinolin-9-yl}amino)heptyl]acridine-9-carboxamide (**2f**)

9-acridinecarboxylic acid (0.202 g, 0.9 mmol) was added to 10 mL THF and dissolved. The ixture was stirred in an ice bath for 2 h. Next, CDMT (0.159 g, 0.9 mmol) and N-methylmorpholine (0.099 mL, 0.9 mmol) was added to the mixture and stirred in an ice bath for another 4 h. Then, once dissolved in 3 mL of THF, compound **1f** (0.269 g, 0.9 mmol) was quickly added to the mixture and stirred continuously for 24 h.

Compound **2f**: yellow oil (yield: 7.87%); FTIR-ATR ν (cm^−1^): 752.5; 1265.2; 1367.5; 1456.2; 1567.6; 2918.2; 3220.5; ^1^H NMR (600 MHz, Methanol-*d*_4_) δ 8.21 (dd, *J* = 8.8 Hz, 2H, ArH), 8.13 (d, *J* = 8.1 Hz, 1H, ArH), 8.07 (dd, *J* = 8.6 Hz, 2H, ArH), 7.84–7.88 (m, 2H, ArH), 7.75 (d, *J* = 7.6 Hz, 1H, ArH), 7.61–7.65 (m, 3H, ArH), 7.43 (t, *J* = 7.7 Hz, 1H, ArH), 3.70–3.73 (m, 2H, CH_2_), 3.66 (t, *J* = 7.0 Hz, 2H, CH_2_), 3.28–3.32 (m, 2H, CH_2_), 3.01 (t, *J* = 7.8 Hz, 2H, CH_2_), 2.17 (p, *J* = 7.6 Hz, 2H, CH_2_), 1.78 (dp, *J* = 14.1, 7.0 Hz, 4H, CH_2_), 1.54–1.59 (m, 4H, CH_2_), 1.33–1.41 (m, 2H, CH_2_); ^13^C NMR (151 MHz, MeOD) δ 167.7, 163.4, 148.2, 142.6, 130.8, 130.8, 128.2, 126.7, 125.1, 125.1, 122.3, 122.0, 121.8, 112.2, 44.5, 39.5, 32.1, 31.0, 31.0, 29.4, 28.9, 28.6, 26.6, 26.2, 22.5, 22.4; MS (ESI) *m*/*z*: 151.0, 185.1, 206.0, 281.2, 324.2; MS-HR (ESI) calcd for C_33_H_34_N_4_O: 502.2761, found: 502.2732.

##### N-[8-({1H,2H,3H-cyclopenta[b]quinolin-9-yl}amino)octyl]acridine-9-carboxamide (**2g**)

9-acridinecarboxylic acid (0.217 g, 0.97 mmol) was added to 10 mL THF and dissolved. The mixture was stirred in an ice bath for 2 h. Next, CDMT (0.171 g, 0.97 mmol) and N-methylmorpholine (0.107 mL, 0.97 mmol) was added to the mixture and stirred in an ice bath for another 4 h. Then, once dissolved in 3 mL of THF, compound **1g** (0.303 g, 0.97 mmol) was quickly added to the mixture and stirred continuously for 24 h.

Compound **2g**: yellow oil (yield: 6.70%); FTIR-ATR ν (cm^−1^): 753.8; 1260.1; 1358.3; 1457.2; 1566.9; 2924.7; 3239.3; ^1^H NMR (600 MHz, Methanol-*d*_4_) δ 8.22 (dd, *J* = 8.8 Hz, 2H, ArH), 8.06–8.10 (m, 3H, ArH), 7.86–7.91 (m, 2H, ArH), 7.74 (d, *J* = 7.7 Hz, 1H, ArH), 7.65–7.69 (m, 2H, ArH), 7.55–7.59 (m, 1H, ArH), 7.38 (t, *J* = 8.2 Hz, 1H, ArH), 3.62–3.69 (m, 4H, CH_2_), 3.27 (t, *J* = 7.2 Hz, 2H, CH_2_), 2.99 (t, *J* = 7.8 Hz, 2H, CH_2_), 2.15 (p, *J* = 7.6 Hz, 2H, CH_2_), 1.75 (dp, *J* = 31.4, 7.8, 7.3 Hz, 4H, CH_2_), 1.46–1.56 (m, 6H, CH_2_), 1.33–1.40 (m, 2H, CH_2_); ^13^C NMR (151 MHz, MeOD) δ 167.7, 166.8, 146.0, 142.6, 130.8, 128.7, 128.2, 126.7, 125.7, 125.2, 123.8, 122.3, 121.0, 112.3, 53.6, 40.4, 33.5, 30.9, 30.8, 29.0, 29.0, 28.9, 28.9, 28.8, 26.7, 26.4, 26.3, 26.3, 22.7; MS (ESI) *m*/*z*: 185.1, 206.0, 295.2, 338.2; MS-HR (ESI) calcd for C_34_H_36_N_4_O: 516.2928, found: 516.2889.

##### N-[9-({1H,2H,3H-cyclopenta[b]quinolin-9-yl}amino)nonyl]acridine-9-carboxamide (**2h**)

9-acridinecarboxylic acid (0.215 g, 0.96 mmol) was added to 10 mL THF and dissolved. The mixture was stirred in an ice bath for 2 h. Next, CDMT (0.169 g, 0.96 mmol) and N-methylmorpholine (0.106 mL, 0.96 mmol) was added to the mixture and stirred in an ice bath for another 4 h. Then, once dissolved in 3 mL of THF, compound **1h** (0.313 g, 0.96 mmol) was quickly added to the mixture and stirred continuously for 24 h.

Compound **2h**: orange oil (yield: 7.35%); FTIR-ATR ν (cm^−1^): 753.3; 1260.0; 1368.3; 1457.2; 1567.3; 2920.4; 3192.7; ^1^H NMR (600 MHz, Methanol-*d*_4_) δ 8.23 (d, *J* = 8.9 Hz, 1H, ArH), 8.07–8.11 (m, 3H, ArH), 7.90–7.94 (m, 1H, ArH), 7.73–7.76 (m, 2H, ArH), 7.68–7.72 (m, 1H, ArH), 7.58–7.61 (m, 2H, ArH), 7.39–7.44 (m, 2H, ArH), 3.64–3.67 (m, 2H, CH_2_), 3.25–3.30 (m, 2H, CH_2_), 3.14 (t, *J* = 7.1 Hz, 2H, CH_2_), 3.01 (t, *J* = 7.8 Hz, 2H, CH_2_), 2.18 (p, *J* = 7.7 Hz, 2H, CH_2_), 1.68 (p, *J* = 7.6 Hz, 2H, CH_2_), 1.31–1.52 (m, 12H, CH_2_); ^13^C NMR (151 MHz, MeOD) δ 172.2, 147.2, 142.6, 130.9, 128.2, 128.2, 126.7, 125.2, 123.5, 122.3, 120.8, 112.3, 53.6, 40.4, 30.9, 30.9, 30.8, 29.2, 29.1, 29.0, 29.0, 29.0, 28.9, 28.8, 28.8, 26.7, 26.4, 26.3, 26.3, 22.8; MS (ESI) *m*/*z*: 206.0, 309.2, 352.2; MS-HR (ESI) calcd for C_35_H_38_N_4_O: 530.3071, found: 530.3045.

#### 4.1.2. General Procedure of Compound **3a**–**3h** Synthesis

To obtain final compounds **3a**–**3h** in form of hydrochlorides, series of compounds **2a**–**2h** were dissolved in methanol (1 mL). Then, 10 mL of HCl/ether was added and stirred for 2 min. After 24 h, the formed precipitate was filtered and dried under the pump with reduced pressure. A physical and elemental analysis was performed for each compound.

##### N-[2-({1H,2H,3H-cyclopenta[b]quinolin-9-yl}amino)ethyl]acridine-9-carboxamide hydrochloride (**3a**)

To the dissolved in (1 mL) methanol, compound **2a** (0.079 g, 0.184 mmol) was added HCl/ether (10 mL) and stirred for 2 min. After 24 h, the formed precipitate was filtered and dried under the pump with reduced pressure. The obtained compound **3a** was an intense yellow solid (82.57% yield) which chars at 250 °C; FTIR-ATR ν (cm^−1^): 755.9; 1243.1; 1370.9; 1465.8; 1569.5; 3023.1; 3232.9; ^1^H NMR (600 MHz, Methanol-*d*_4_) δ 8.50 (d, *J* = 8.4 Hz, 1H, ArH), 8.43 (dd, *J* = 8.8 Hz, 2H, ArH), 8.34–8.39 (m, 2H, ArH), 8.32 (dd, *J* = 8.7 Hz, 2H, ArH), 7.93 (t, *J* = 8.2 Hz, 1H), 7.84–7.89 (m, 3H, ArH), 7.71 (t, *J* = 8.3 Hz, 1H, ArH), 4.35 (t, *J* = 6.2 Hz, 2H, CH_2_), 4.14 (t, *J* = 6.2 Hz, 2H, CH_2_), 3.52 (q, *J* = 7.0 Hz, 2H, CH_2_), 3.27 (t, *J* = 7.9 Hz, 2H, CH_2_), 2.36 (p, *J* = 7.7 Hz, 2H, CH_2_); ^13^C NMR (151 MHz, MeOD) δ 165.4, 152.6, 140.3, 137.9, 132.7, 128.9, 126.7, 126.5, 122.6, 122.6, 119.8, 119.4, 117.2, 44.4, 40.3, 31.2, 22.5; MS (ESI) *m*/*z*: 185.1, 206.0, 226.1, 249.1; MS-HR (ESI) calcd for C_28_H_24_N_4_O: 432.1950, found: 432.1960.

##### N-[3-({1H,2H,3H-cyclopenta[b]quinolin-9-yl}amino)propyl]acridine-9-carboxamide hydrochloride (**3b**)

To the dissolved in (1 mL) methanol, compound **2b** (0.051 g, 0.114 mmol) was added HCl/ether (10 mL) and stirred for 2 min. After 24 h, the formed precipitate was filtered and dried under the pump with reduced pressure. Obtained compound **3b** was intense yellow solid (64.00% yield) which chars at 200 °C; FTIR-ATR ν (cm^−1^): 753.4; 1260.6; 1370.2; 1464.6; 1558.3; 3027.4; 3222.9; ^1^H NMR (600 MHz, Methanol-*d*_4_) δ 8.47 (d, *J* = 8.5 Hz, 1H, ArH), 8.41–8.45 (m, 4H, ArH), 8.36–8.39 (m, 2H, ArH), 8.01–8.04 (m, 2H, ArH), 7.90 (t, *J* = 8.0 Hz, 1H, ArH), 7.80 (d, *J* = 8.2 Hz, 1H, ArH), 7.67 (t, *J* = 8.2 Hz, 1H, ArH), 4.12 (t, *J* = 7.1 Hz, 2H, CH_2_), 3.91 (t, *J* = 7.0 Hz, 2H, CH_2_), 3.52 (q, *J* = 6.7 Hz, 2H, CH_2_), 3.24 (t, *J* = 7.9 Hz, 2H, CH_2_), 2.29–2.36 (m, 4H, CH_2_); ^13^C NMR (151 MHz, MeOD) δ 165.0, 140.7, 137.7, 137.5, 132.5, 128.9, 126.8, 126.2, 122.7, 120.3, 119.2, 117.2, 42.3, 37.2, 31.3, 31.2, 30.7, 22.4; MS (ESI) *m*/*z*: 185.1, 223.1, 235.1, 263.1; MS-HR (ESI) calcd for C_29_H_26_N_4_O: 446.2107, found: 446.2106.

##### N-[4-({1H,2H,3H-cyclopenta[b]quinolin-9-yl}amino)butyl]acridine-9-carboxamide hydrochloride (3**c**)

To the dissolved in (1 mL) methanol, compound **2c** (0.064 g, 0.138 mmol) was added HCl/ether (10 mL) and stirred for 2 min. After 24 h, the formed precipitate was filtered and dried under the pump with reduced pressure. Obtained compound **3c** was intense yellow solid (74.06% yield) which chars at 240 °C; FTIR-ATR ν (cm^−1^): 743.9; 1263.7; 1368.4; 1466.9; 1552.4; 3025.9; 3189.1; ^1^H NMR (600 MHz, Methanol-*d*_4_) δ 8.42–8.47 (m, 3H, ArH), 8.36–8. 41 (m, 4H, ArH), 7.99–8.03 (m, 2H, ArH), 7.88 (t, *J* = 8.2 Hz, 1H, ArH), 7.78 (d, *J* = 7.9 Hz, 1H, ArH), 7.67 (t, *J* = 8.3 Hz, 1H, ArH), 4.00 (t, *J* = 6.6 Hz, 2H, CH_2_), 3.82 (t, *J* = 6.5 Hz, 2H, CH_2_), 3.48 (q, *J* = 7.3, 6.2 Hz, 2H, CH_2_), 3.22 (t, *J* = 7.9 Hz, 2H, CH_2_), 2.31 (p, *J* = 7.7 Hz, 2H, CH_2_), 1.97–2.05 (m, 4H, CH_2_); ^13^C NMR (151 MHz, MeOD) δ 164.5, 161.1, 153.4, 140.3, 137.8, 137.6, 132.5, 128.9, 126.9, 126.2, 122.7, 122.5, 119.9, 119.2, 112.3, 44.1, 39.6, 31.3, 31.1, 28.7, 26.1, 22.4; MS (ESI) *m*/*z*: 185.1, 206.0, 239.2, 282.2; MS-HR (ESI) calcd for C_30_H_28_N_4_O: 460.2263, found: 460.2276.

##### N-[5-({1H,2H,3H-cyclopenta[b]quinolin-9-yl}amino)pentyl]acridine-9-carboxamide hydrochloride (**3d**)

To the dissolved in (1 mL) methanol, compound **2d** (0.076 g, 0.16 mmol) was added HCl/ether (10 mL) and stirred for 2 min. After 24 h, the formed precipitate was filtered and dried under the pump with reduced pressure. Obtained compound **3d** was intense yellow solid (86.49% yield) which chars at 240 °C; FTIR-ATR ν (cm^−1^): 748.1; 1267.4; 1369.7; 1465.6; 1557.9; 3030.0; 3185.2; ^1^H NMR (600 MHz, Methanol-*d*_4_) δ 8.43–8.47 (m, 2H, ArH), 8.41–8.36 (m, 5H, ArH), 7.99–8.04 (m, 2H, ArH), 7.86 (t, *J* = 8.2 Hz, 1H, ArH), 7.76 (d, *J* = 7.9 Hz, 1H, ArH), 7.61 (t, *J* = 8.3 Hz, 1H, ArH), 3.93 (t, *J* = 7.3 Hz, 2H, CH_2_), 3.78 (t, *J* = 7.1 Hz, 2H, CH_2_), 3.46 (t, *J* = 7.2 Hz, 2H, CH_2_), 3.22 (t, *J* = 7.9 Hz, 2H, CH_2_), 2.32 (p, *J* = 7.7 Hz, 2H, CH_2_), 1.95 (dp, *J* = 14.6, 7.4 Hz, 4H, CH_2_), 1.72 (p, *J* = 7.7 Hz, 2H, CH_2_); ^13^C NMR (151 MHz, MeOD) δ 164.4, 153.3, 140.2, 137.9, 137.6, 132.4, 128.9, 126.9, 126.1, 122.7, 122.5, 119.8, 119.2, 117.1, 44.4, 39.8, 31.3, 31.1, 30.6, 28.8, 23.8, 22.3; MS (ESI) *m*/*z*: 185.1, 206.1, 268.2, 296.2; MS-HR (ESI) calcd for C_31_H_30_N_4_O: 474.2420, found: 474.2441.

##### N-[6-({1H,2H,3H-cyclopenta[b]quinolin-9-yl}amino)hexyl]acridine-9-carboxamide hydrochloride (**3e**)

To the dissolved in (1 mL) methanol, compound **2e** (0.039 g, 0.081 mmol) was added HCl/ether (10 mL) and stirred for 2 min. After 24 h, the formed precipitate was filtered and dried under the pump with reduced pressure. Obtained compound **3e** was intense yellow solid (62.5% yield) which melts with chars at 160 °C; FTIR-ATR ν (cm^−1^): 753.7; 1262.9; 1368.9; 1464.3; 1558.2; 2934.2; 3196.3; ^1^H NMR (600 MHz, Methanol-*d*_4_) δ 8.44–8.47 (m, 2H, ArH), 8.36–8.42 (m, 5H, ArH), 8.02–8.05 (m, 2H, ArH), 7.87 (t, *J* = 8.2 Hz, 1H, ArH), 7.76 (d, *J* = 7.9 Hz, 1H, ArH), 7.64 (t, *J* = 8.3 Hz, 1H, ArH), 3.91–3.88 (m, 2H, CH_2_), 3.75 (t, *J* = 7.2 Hz, 2H, CH_2_), 3.44 (t, *J* = 7.3 Hz, 2H, CH_2_), 3.21 (t, *J* = 7.9 Hz, 2H, CH_2_), 2.32 (p, *J* = 7.8 Hz, 2H, CH_2_), 1.85–1.92 (m, 4H, CH_2_), 1.65 (p, *J* = 3.5 Hz, 4H, CH_2_); ^13^C NMR (151 MHz, MeOD) δ 164.3, 153.3, 140.2, 137.9, 137.6, 132.4, 128.9, 126.9, 126.1, 122.7, 122.5, 119.8, 119.2, 117.1, 44.5, 39.8, 39.2, 31.3, 31.1, 31.0, 28.9, 28.8, 26.5, 26.2, 25.8, 20.9; MS (ESI) *m*/*z*: 180.1, 206.1, 267.2, 310.2; MS-HR (ESI) calcd for C_32_H_32_N_4_O: 488.2576, found: 488.2600.

##### N-[7-({1H,2H,3H-cyclopenta[b]quinolin-9-yl}amino)heptyl]acridine-9-carboxamide hydrochloride (**3f**)

To the dissolved in (1 mL) methanol, compound **2f** (0.036 g, 0.071 mmol) was added HCl/ether (10 mL) and stirred for 2 min. After 24 h, the formed precipitate was filtered and dried under the pump with reduced pressure. Obtained compound **3f** was intense yellow solid (78.64% yield) which melts and chars at 180 °C; FTIR-ATR ν (cm^−1^): 753.5; 1263.9; 1362.6; 1464.4; 1558.4; 2931.3; 3196.4; ^1^H NMR (600 MHz, Methanol-*d*_4_) δ 8.44–8.47 (m, 2H, ArH), 8.35–8.40 (m, 5H, ArH), 8.00–8.04 (m, 2H, ArH), 7.85–7.88 (m, 1H, ArH), 7.76 (d, *J* = 7.8 Hz, 1H, ArH), 7.64 (t, *J* = 7.8 Hz, 1H, ArH), 3.85–3.89 (m, 2H, CH_2_), 3.74 (t, *J* = 7.2 Hz, 2H, CH_2_), 3.43 (t, *J* = 7.3 Hz, 2H, CH_2_), 3.20 (t, *J* = 7.9 Hz, 2H, CH_2_), 2.32 (p, *J* = 7.8 Hz, 2H, CH_2_), 1.85 (q, *J* = 6.9 Hz, 4H, CH_2_), 1.60 (d, *J* = 15.0 Hz, 6H, CH_2_); ^13^C NMR (151 MHz, MeOD) δ 164.3, 153.3, 153.3, 140.3, 137.8, 137.6, 132.4, 128.9, 126.8, 126.1, 122.7, 122.5, 119.8, 119.2, 117.1, 44.5, 39.8, 31.2, 31.1, 31.0, 28.9, 28.6, 26.7, 26.2, 22.3; MS (ESI) *m*/*z*: 178.1, 206.1, 296.2, 324.2; MS-HR (ESI) calcd for C_33_H_34_N_4_O: 502.2733, found: 502.2749.

##### N-[8-({1H,2H,3H-cyclopenta[b]quinolin-9-yl}amino)octyl]acridine-9-carboxamide hydrochloride (**3g**)

To the dissolved in (1 mL) methanol, compound **2g** (0.034 g, 0.065 mmol) was added HCl/ether (10 mL) and stirred for 2 min. After 24 h, the formed precipitate was filtered and dried under the pump with reduced pressure. Obtained compound **3g** was intense yellow solid (91.29% yield) which chars at 185 °C; FTIR-ATR ν (cm^−1^): 753.6; 1262.6; 1362.5; 1464.4; 1558.3; 2927.6; 3196.6; ^1^H NMR (600 MHz, Methanol-*d*_4_) δ 8.43–8.46 (m, 2H, ArH), 8.34–8.40 (m, 5H, ArH), 8.01–8.05 (m, 2H, ArH), 7.85 (t, *J* = 7.2 Hz, 1H, ArH), 7.75 (d, *J* = 8.0 Hz, 1H, ArH), 7.63 (t, *J* = 7.3 Hz, 1H, ArH), 3.83–3.88 (m, 2H, CH_2_), 3.73 (t, *J* = 7.2 Hz, 2H, CH_2_), 3.42 (t, *J* = 7.2 Hz, 2H, CH_2_), 3.21 (t, *J* = 7.9 Hz, 2H, CH_2_), 2.32 (p, *J* = 7.8 Hz, 2H, CH_2_), 1.84 (p, *J* = 7.1 Hz, 4H, CH_2_), 1.48–1.61 (m, 8H, CH_2_); ^13^C NMR (151 MHz, MeOD) δ 164.4, 153.3, 153.1, 140.4, 137.7, 137.6, 132.4, 128.9, 126.8, 126.0, 122.6, 122.4, 119.9, 119.2, 117.1, 44.6, 39.9, 31.2, 31.1, 31.0, 29.0, 28.9, 28.8, 26.7, 26.2, 22.3; MS (ESI) *m*/*z*: 185.1, 206.1, 295.2, 338.2; MS-HR (ESI) calcd for C_34_H_36_N_4_O: 516.2889, found: 516.2902.

##### N-[9-({1H,2H,3H-cyclopenta[b]quinolin-9-yl}amino)nonyl]acridine-9-carboxamide hydrochloride (**3h**)

To the dissolved in (1 mL) methanol, compound **2h** (0.038 g, 0.071 mmol) was added HCl/ether (10 mL) and stirred for 2 min. After 24 h, the formed precipitate was filtered and dried under the pump with reduced pressure. Obtained compound **3h** was intense yellow solid (89.66% yield); mp 190–195 °C; FTIR-ATR ν (cm^−1^): 753.7; 1262.6; 1362.6; 1464.4; 1558.4; 2923.5; 3196.6; ^1^H NMR (600 MHz, Methanol-*d*_4_) δ 8.43–8.46 (m, 2H, ArH), 8.33–8.40 (m, 5H, ArH), 8.01–8.05 (m, 2H, ArH), 7.86 (t, *J* = 7.2 Hz, 1H, ArH), 7.75 (d, *J* = 8.4 Hz, 1H, ArH), 7.63 (t, *J* = 8.2 Hz, 1H, ArH), 3.83–3.87 (m, 2H, CH_2_), 3.73 (t, *J* = 7.2 Hz, 2H, CH_2_), 3.41 (t, *J* = 7.3 Hz, 2H, CH_2_), 3.20 (t, *J* = 7.9 Hz, 2H, CH_2_), 2.32 (p, *J* = 7.7 Hz, 2H, CH_2_), 1.78–1.87 (m, 4H, CH_2_), 1.59–1.44 (m, 12H, CH_2_); ^13^C NMR (151 MHz, MeOD) δ 164.4, 153.3, 153.1, 140.4, 137.7, 132.4, 128.9, 126.8, 126.0, 122.6, 122.4, 120.0, 119.1, 117.1, 44.6, 39.9, 31.2, 31.1, 31.0, 29.2, 29.0, 28.9, 28.8, 26.8, 26.2, 22.3; MS (ESI) *m*/*z*: 185.1, 206.0, 223.1, 253.2, 280.2, 309.2, 336.2, 352.2; MS-HR (ESI) calcd for C_35_H_38_N_4_O: 530.3046, found: 530.3072.

### 4.2. Biological Evaluation

#### 4.2.1. In Vitro Inhibition Studies on AChE and BuChE

An evaluation of the inhibitory potency towards AChE (EC 3.1.1.7, *Electrophorus electricus*) and BuChE (EC 3.1.1.8, equine serum) was performed using a modified spectrophotometric Ellman’s method [29,34]. All necessary reagents for the study were purchased from Sigma Aldrich (Steinheim, Germany). As references, the compounds tacrine and bistacrine were used. The phosphate buffer (pH 8.0) was used as a solvent for reagents. The assay protocol included 14 µL of the tested compound in the nine different concentrations, and 40 µL of substrate acetylthiocholine (ATI) in concentration 1mM or 2mM for AChE and BuChE, respectively. Next, 76 µL of 0.4 mg/mL 5,5′-dithio-bis(2-nitrobenzoic) acid (DTNB) was added to the mixture. In the end, to start the reaction 10 µL of AChE (2U/mL) or BuChE (4U/mL) was added and the solution was mixed and incubated for 10 min. at 37 °C. As a control, instead of the tested compound, we used PBS. The measurements of absorbance were recorded at 412 nm in 96-well microplates using the spectrophotometric plate reader (Synergy H1, Biotek, Santa Clara, CA, USA). The experiments were performed independently and in triplicate. Data were expressed as mean ± SD.

#### 4.2.2. Kinetic Characterization of AChE and BuChE Inhibition

The most potent compounds with the highest inhibitory activity were selected for the kinetic studies. The protocol of the kinetic study was similar to the previous inhibition study. A phosphate buffer (pH 8.0) was used as the solvent for all reagents. The tested medium consisted of 76 µL DTNB (0.4 mg/mL), 40 µL substrate (ATI) at chosen concentrations (35–350 µM), 10 µL of AChE (2 U/mL) or BuChE (4 U/mL), and 14 µL of the inhibitor. The kinetic characterization of AChE inhibition by the most potent derivative against AChE, **3f** was performed using four concentrations of compound (0, 200, 400, 800 nM). For the BuChE kinetic analysis, we used four concentrations of the **3b** derivative (0, 150, 200, 250 nM). Changes in absorbance were measured at 412 nm. All experiments were conducted independently and in triplicate. To estimate the type of inhibition, reciprocal plots of 1/V versus 1/[S] were created [36].

#### 4.2.3. Antioxidant Activity Evaluation

##### ORAC-FL Assay

The antioxidant activity of the selected compounds was measured using an Oxygen Radical Absorbance Capacity-Fluorescein (ORAC-FL) assay according to the methodology of Ou et al. [43] with modifications described by Davalos et al. [44]. Fluorescein disodium salt (FL), 2,2′-azobis(2-methylpropionamidine) dihydrochloride (APPH), and 6-hydroxy-2,5,7,8-tetramethyl-chromane-2-carboxylic acid (Trolox^®^) as a standard was purchased from Sigma Aldrich (Steinheim, Germany). The assay was carried out in 75 mM phosphate buffer (pH 7.4) with the final reaction volume 200 µL. All reagents were dissolved in 75 mM phosphate buffer (pH 7.4). The final concentrations were 1–13 μM for both Trolox and compounds. A solution of the tested compounds/Trolox (20 µL), and fluorescein (120 µL, 70 nM final concentration), were placed in the well of the black 96 well-microplate (Greiner Bio-One, Frickenhausen, Germany). The mixture was preincubated in the dark at 37 °C for 15 min. After incubation, the reaction was initiated by the quick addition of 60 µL of APPH (12 mM, final concentration) using a multichannel pipette. The plate was immediately placed in a multifunctional microplate reader (Synergy H1, BioTek). Measurements of the fluorescence from the top were performed kinetically at 70 s intervals for 140 min at 37 °C using a microplate reader. Shifts of fluorescence were measured at 485 nm excitation and 520 nm emission using the fluorescent monochromators. As a blank (FL + APPH) was used 75 mM phosphate buffer (pH 7.4) instead of the tested compounds/Trolox. The fluorescein stability during measurements was assessed by addition 75 mM phosphate buffer (pH 7.4) instead of the tested compounds/Trolox and APPH. The plate was automatically shaken before every measurement with a maximum intensity of 10 s. All tests were performed in triplicate and at least three independent runs for each sample.

Raw data were exported from the Gen5 Data Analysis to MS Excel for further calculations. Curves of the compounds and Trolox^®^ (fluorescence versus time) were firstly normalized to the curve of the blank for the same assay by multiplying fluorescence data by the factor (fluorescence_blank,*t*=0_/fluorescence_sample,*t*=0_). The area under the fluorescence decay curve (*AUC*) was calculated using equation:

(1) $$AUC=1+\overset{i=86}{\underset{i=1}{\sum }}f_{i}/f_{0}$$

(2) $$Net\;AUC=AUC_{sample}-AUC_{blank}$$

The *Net AUC* values for each compound and Trolox^®^ were calculated according to the equation and plotted against their concentrations. Trolox^®^ equivalents were determined by the ratio of the slope of the linear regression curve of each compound with the slope of a standard curve. Data are expressed as mean ± SD.

(3) $$TE\;\left(range\;of\;concentrations\right)=m_{compound}/m_{Trolox}$$

#### 4.2.4. Hepatotoxicity Assay

Human hepatic stellate cells (HSCs, Sciencell, Carlsbad, CA, USA) were grown in a dedicated medium (Stellate Cell Medium, Sciencell) with 1% Stellate Cell Growth Supplement, 2% fetal bovine serum (FBS), and 1% penicillin/streptomycin solution (Sciencell). Cells were kept in an incubator (37 °C, 5% CO_2_). At the start of the experiment, cells were plated in 96-well plates at a density of 5 × 10^3^ cells per well and incubated for 24 h. After the incubation period, the medium was removed and the cells were exposed to 100 µL of compound solutions in the concentration range (10–0.1 µM) or medium (blank). Cells were further incubated for 24 h. Finally, the medium was removed, the cells were washed with PBS, and 50 µL of MTT solution (0.75 mg/mL) was added to the cells. The plates were kept in the dark for 2 h at 37 °C. Finally the MTT solution was removed and 100 µL of DMSO was added to each well. The plates with DMSO were kept at room temperature for 10 min. After this time, 5 µL of Sorensen Buffer was added to each well. The plates were shaken and the absorbance was measured at 570 nm using a microplate reader (Synergy H1, BioTek). The results were presented as IC_50_ values, representing the inhibitory concentration which reduces the cells’ viability to 50% compare to control (100% viability) [36,45,46,47].

#### 4.2.5. Molecular Modeling

The molecule builder-editor, implemented in the Avogadro 1.2.0 software, was used for creation of three-dimensional structures of the set of investigated ligands **3a**–**3h** [48]. Density functional theory (DFT) method at B3LYP/6–311++G (d,p) level of theory, implemented in the Gaussian 09 package, was employed to gain insights into the molecular structure of the studied ligands (e.g., the lowest energy conformation and the molecular electrostatic potential) as well as to assess the flexibility of the aliphatic linker of the those compounds [49]. The molecular docking has been performed using two cross-checked methods, implemented in Flare 5.0.0. platform and in AutoDockVina 1.2.0 program [50,51]. The AChE and BuChE models have been based on the experimentally resolved PDB id: 7D9O and 7BGC crystal structures, respectively [52,53]. The preparation AChE and BuChE models for docking, including cleaning the protein structures, adding hydrogens, protonating histidine’s’ Nε atoms, removing ligands and water molecules etc., was done using the protein-preparation tools implemented in Flare platform [50]. The template ligand and water molecules were removed. The active site of both enzymes included residues within the cut-off of 10 Å from the original ligands which had been resolved for the experimental crystal structures. The docking calculations were done using the Extra Precision Docking Algorithm implemented in Flare as well as Iterated Local Search global optimizer approach implemented in AutoDockVina [50,51]. The results of the best ligand positions with the lowest docking score obtained from Flare calculations were then cross-checked with the corresponding results of minimum docking energy obtained with AutoDockVina. The poses which were evaluated as the best positions of ligands in both approaches have been chosen as the final ligands’ poses. The results were visualized and analyzed using VMD 1.9.3 software [54].

#### 4.2.6. ADMET Analysis

All structures in tested series of compounds were analyzed by using ACDLabs Percepta software version 14.0.0 (Advanced Chemistry Development, Inc., Metropolitan Toronto, ON, Canada), SwissADME service (Swiss Institute of Bioinformatics 2021) [55], admetSAR 2.0 service (admetSAR 2019) [56] and ProTOX II service [57] to determine the computational pharmacokinetic and toxicological profiles of tested compounds.

## 5. Conclusions

The novel series of cyclopentaquinoline and 9-acridinecarboxylic acid presented significant activity towards both AChE and BuChE. The interplay of the abovementioned factors results in the outcome ligand’s affinity towards an enzyme. The simultaneous interactions of the acridine and cyclopentaquinoline moieties with the key aromatic ligand-binding residues inside the enzyme’s pocket (i.e., dual binding mode) ensure ligand’s affinity, which is proportional to the extent of interactions between a ligand and the residues inside the pocket. The **3b** derivative is the most potent compound according to the results from in vitro biological studies and ADMET predictions. Compound **3b** should be reconsidered for further analysis as a promising drug candidate for the treatment of AD.

## Figures and Tables

**Table 1:** Inhibitory activity of compounds **3a**–**3h** towards AChE and BuChE and radical scavenging ability.

Compound	AChE IC_50_ ± SD [nM] ^a^	BuChE IC_50_ ± SD [nM] ^b^	Selectivity for AChE ^c^	Selectivity for BuChE ^d^	ORAC (TE) ^e^
**3a**	8619.37 ± 606.33	539.92 ± 66.90	0.063	15.96	n.d.
**3b**	272.33 ± 35.11	103.73 ± 7.63	0.38	2.62	0.4614 ± 0.056
**3c**	272.79 ± 22.11	200.20 ± 18.07	0.73	1.36	n.d.
**3d**	4057.16 ± 535.79	173.41 ± 18.89	0.043	23.39	n.d.
**3e**	394.69 ± 36.07	194.88 ± 11.02	0.49	2.02	n.d.
**3f**	113.34 ± 7.14	203.52 ± 16.02	1.79	0.56	0.3254 ± 0.014
**3g**	700.98 ± 67.08	177.63 ± 17.03	0.25	3.95	n.d.
**3h**	669.42 ± 83.39	226.97 ± 27.05	0.34	2.95	n.d.
Tacrine	226.97 ± 27.05	7.2 ± 0.51	0.032	31.52	0.0132 ± 0.009
Bistacrine	405.10 ± 28.62	226 ± 30.40	0.56	1.79	n.d.

^a^ IC_50_: 50% inhibitory concentration (means ± SD of three independent experiments) of AChE. ^b^ IC_50_: 50% inhibitory concentration (means ± SD of three independent experiments) of BuChE. ^c^ Selectivity for AChE: IC_50_(BuChE)/IC_50_(AChE). ^d^ Selectivity for BuChE: IC_50_(AChE)/IC_50_(BuChE). ^e^ Data are expressed as Trolox equivalents (TE) and are shown as mean ± SD. n.d.: not determined.

**Table 2:** Basic physicochemical properties of compounds. Log P value is an average of 5 prediction algorithms (iLOGP, XLOGP, WLOGP, MLOGP, SILICOS-IT). TPSA—Topological Polar Surface Area.

Compound	Molecular Weight [g/mol]	LogP	pKa (acid)	pKa (base)	TPSA [Å^2^]	Molar Refractivity [m^3^/mol]	H-bond Acceptors	H-bond Donors
3a	432.52	4.70	13.19	8.71	66.91	133.80	3	2
3b	446.54	5.03	13.52	8.90	66.91	138.61	3	2
3c	460.57	5.31	13.65	8.97	66.91	143.42	3	2
3d	474.60	5.47	13.71	9.00	66.91	148.23	3	2
3e	488.62	5.93	13.73	9.01	66.91	153.03	3	2
3f	502.65	6.17	13.73	9.02	66.91	157.84	3	2
3g	516.68	6.60	13.74	9.02	66.91	162.65	3	2
3h	530.70	6.96	13.74	9.02	66.91	167.45	3	2

## Data Availability

Not applicable.

## Supplementary Materials

The following are available online at https://www.mdpi.com/article/10.3390/ijms23115876/s1.
