# Supplementary material for: Novel Cyclopentaquinoline and Acridine Analogs as Multifunctional, Potent Drug Candidates in Alzheimer’s Disease
Source: Int J Mol Sci. 2022 May 24;23(11):5876. doi: 10.3390/ijms23115876 (PMC9179981; doi:10.3390/ijms23115876)
Supplement: Supplementary file 1 [file ijms-23-05876-s001.zip › ijms-1730980-supplementary.pdf]

Supplementary materials

Novel cyclopentaquinoline and acridine analogs as multifunctional, potent drug candidates in Alzheimer's disease

Karolina Maciejewska [1], Kamila Czarnecka [1], Paweł Kręcisz [1], Dorota Niedziałek [2], Grzegorz Wieczorek [2], Robert Skibiński [3], and Paweł Szymański [1,4,*]

[1] Department of Pharmaceutical Chemistry, Drug Analyses and Radiopharmacy, Faculty of Pharmacy, Medical University of Lodz, Muszyńskiego 1 St., 90-151 Lodz, Poland; karolina.maciejewska@stud.umed.lodz.pl (K.M.); kamila.czarnecka@umed.lodz.pl (K.C); pawel.krecisz@stud.umed.lodz.pl (P.K.); pawel.szymanski@umed.lodz.pl (P.S.)

[2] Institute of Biochemistry and Biophysics, Polish Academy of Sciences, 02-106 Warsaw, Poland; dorotanka@gmail.com (D.N.); wieczorek.grzegorz@gmail.com (G.W.)

[3] Department of Medicinal Chemistry, Faculty of Pharmacy, Medical University of Lublin, Jaczewskiego 4 St., 20-090 Lublin, Poland; robertskibinski@umlub.pl (R.S.)

[4] Department of Radiobiology and Radiation Protection, Military Institute of Hygiene and Epidemiology, 4 Kozielska St., 01-163 Warsaw, Poland; pawel.szymanski@wihe.pl (P.S.)

* pawel.szymanski@umed.lodz.pl; phone: +48-42-677-92-53

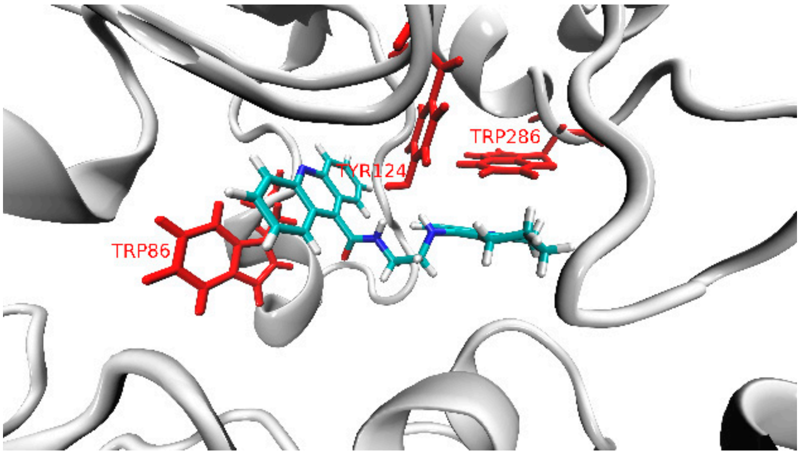

Figure S1. The binding mode of the **3a** ligand inside the pocket of AChE. The key ligand-binding aromatic residues in the pocket of AChE are marked in red.

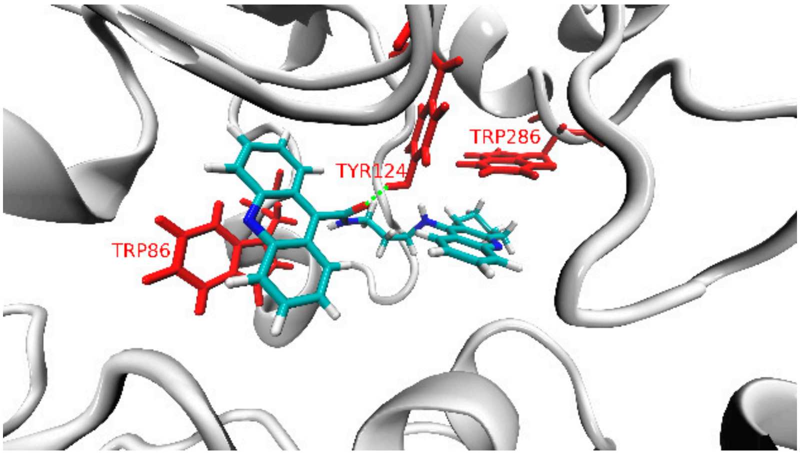

Figure S2. The binding mode of the **3b** ligand inside the pocket of AChE. The key ligand-binding aromatic residues in the pocket of AChE are marked in red.

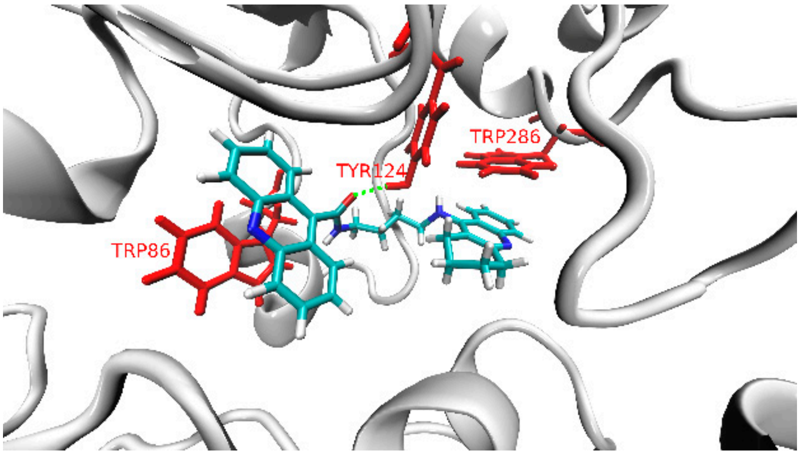

Figure S3. The binding mode of the **3c** ligand inside the pocket of AChE. The key ligand-binding aromatic residues in the pocket of AChE are marked in red.

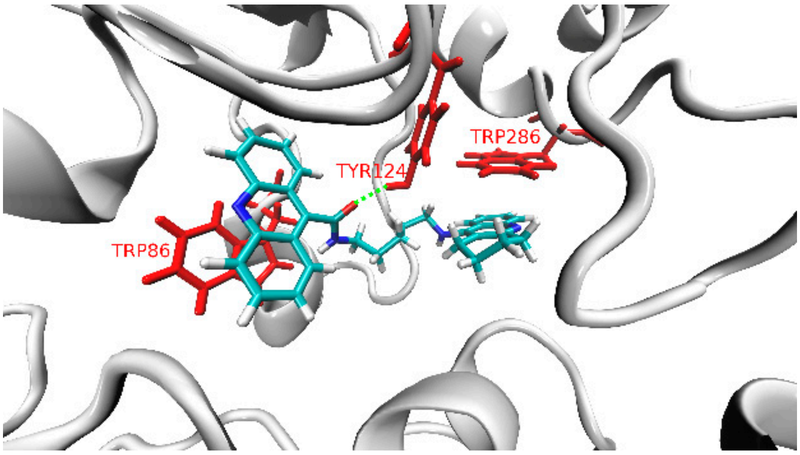

Figure S4. The binding mode of the **3d** ligand inside the pocket of AChE. The key ligand-binding aromatic residues in the pocket of AChE are marked in red.

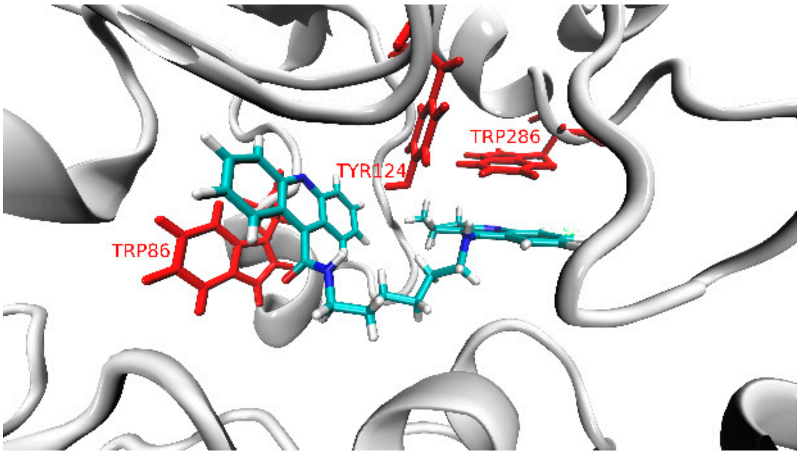

Figure S5. The binding mode of the **3e** ligand inside the pocket of AChE. The key ligand-binding aromatic residues in the pocket of AChE are marked in red.

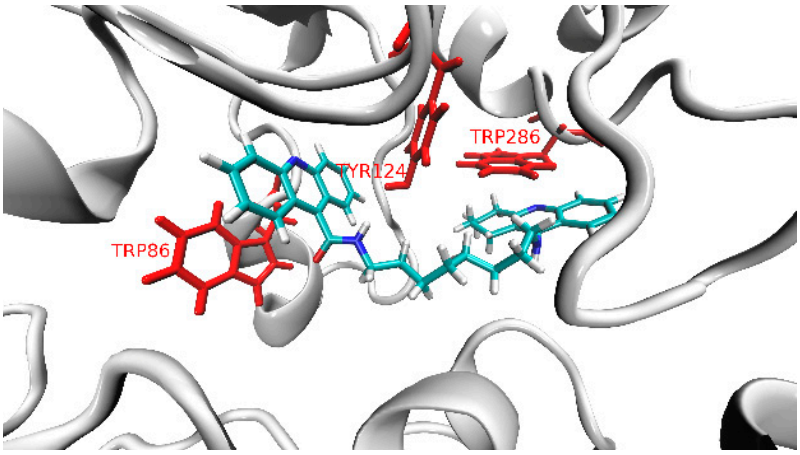

Figure S6. The binding mode of the **3g** ligand inside the pocket of AChE. The key ligand-binding aromatic residues in the pocket of AChE are marked in red.

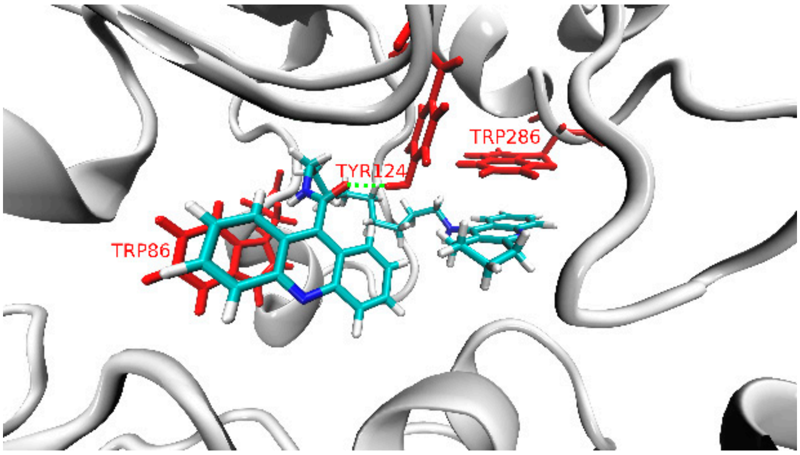

Figure S7. The binding mode of the **3h** ligand inside the pocket of AChE. The key ligand-binding aromatic residues in the pocket of AChE are marked in red.

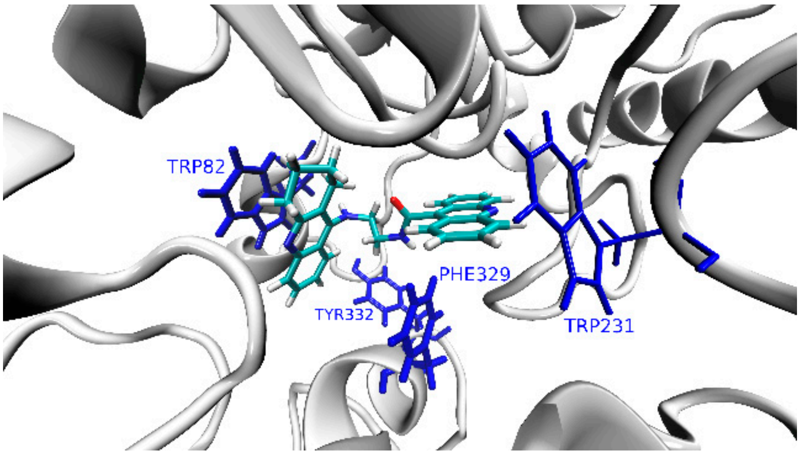

Figure S8. The binding mode of the **3a** ligand inside the pocket of BuChE. The key ligand-binding aromatic residues in the pocket of BuChE are marked in blue.

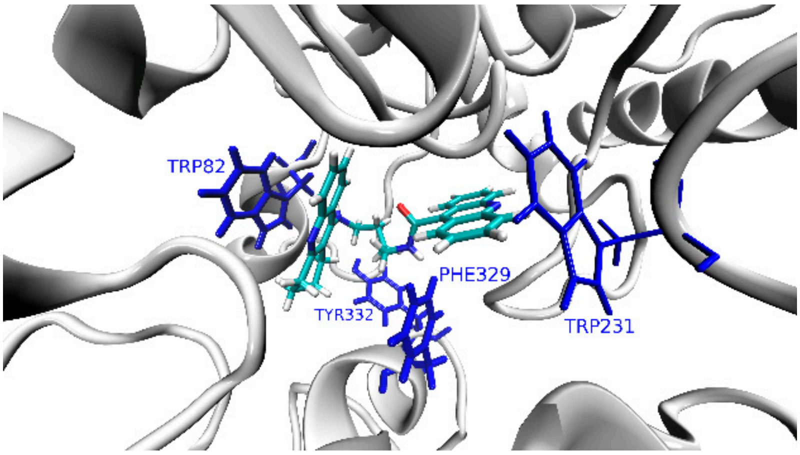

Figure S9. The binding mode of the **3c** ligand inside the pocket of BuChE. The key ligand-binding aromatic residues in the pocket of BuChE are marked in blue.

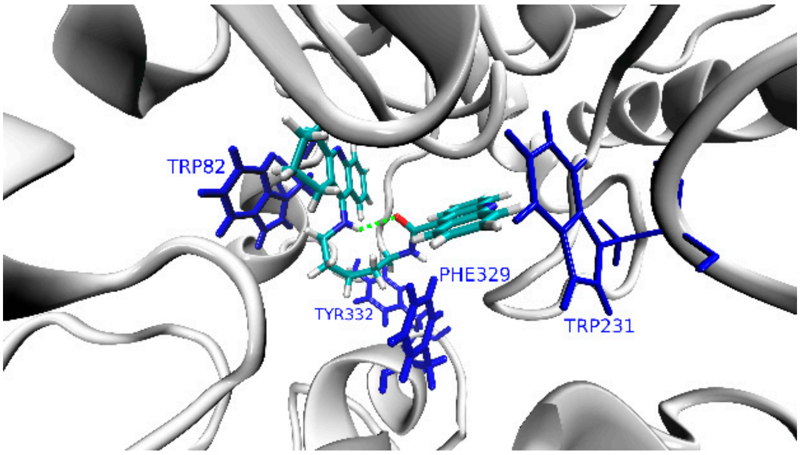

Figure S10. The binding mode of the **3d** ligand inside the pocket of BuChE. The key ligand-binding aromatic residues in the pocket of BuChE are marked in blue.

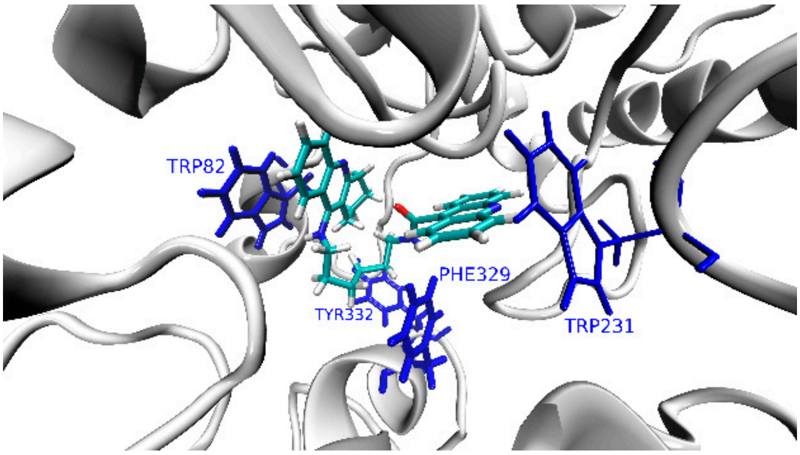

Figure S11. The binding mode of the **3e** ligand inside the pocket of BuChE. The key ligand-binding aromatic residues in the pocket of BuChE are marked in blue.

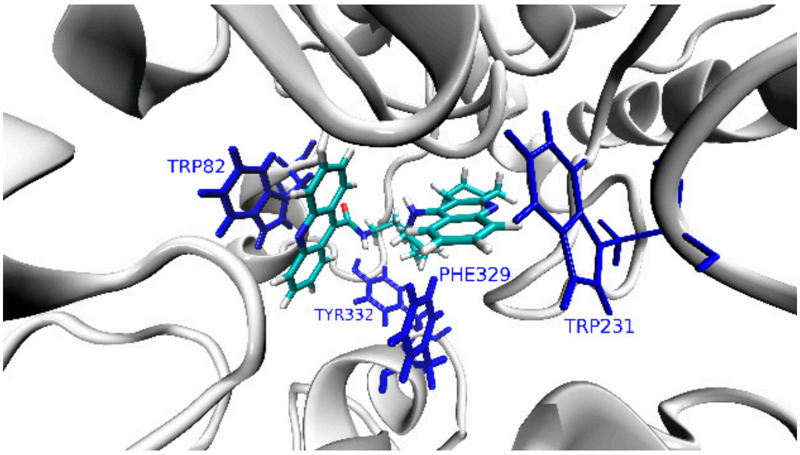

Figure S12. The binding mode of the **3f** ligand inside the pocket of BuChE. The key ligand-binding aromatic residues in the pocket of BuChE are marked in blue.

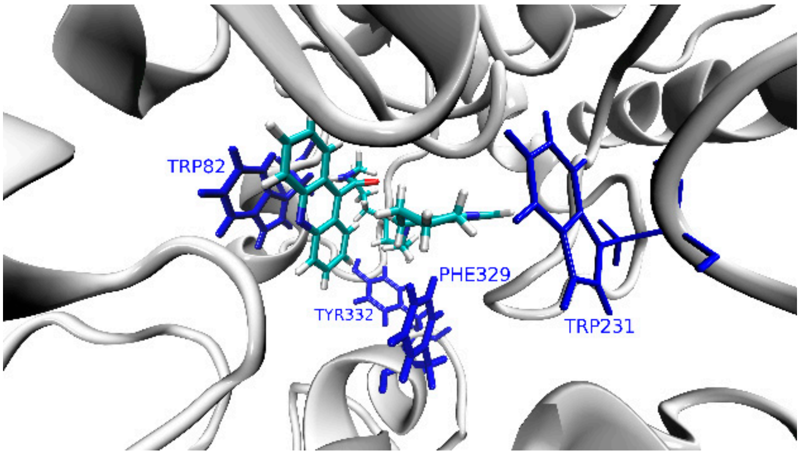

Figure S13. The binding mode of the **3g** ligand inside the pocket of BuChE. The key ligand-binding aromatic residues in the pocket of BuChE are marked in blue.

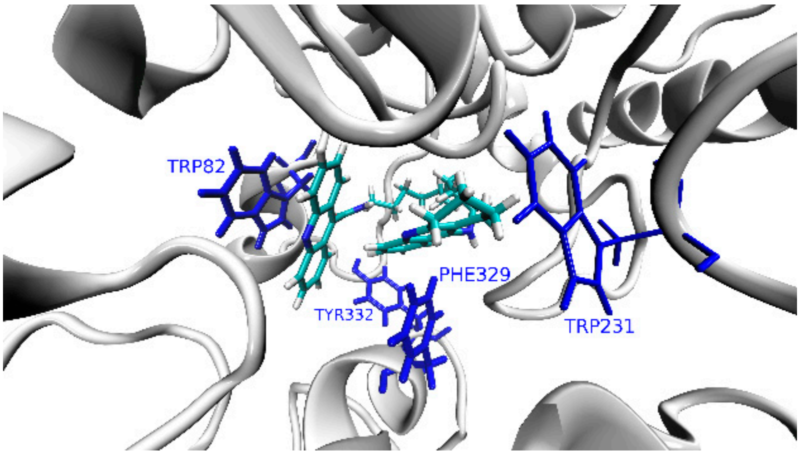

Figure S14. The binding mode of the **3h** ligand inside the pocket of BuChE. The key ligand-binding aromatic residues in the pocket of BuChE are marked in blue.
